# On bias, variance, overfitting, gold standard and consensus in single-particle analysis by cryo-electron microscopy

**DOI:** 10.1107/S2059798322001978

**Published:** 2022-03-16

**Authors:** C. O. S. Sorzano, A. Jiménez-Moreno, D. Maluenda, M. Martínez, E. Ramírez-Aportela, J. Krieger, R. Melero, A. Cuervo, J. Conesa, J. Filipovic, P. Conesa, L. del Caño, Y. C. Fonseca, J. Jiménez-de la Morena, P. Losana, R. Sánchez-García, D. Strelak, E. Fernández-Giménez, F. P. de Isidro-Gómez, D. Herreros, J. L. Vilas, R. Marabini, J. M. Carazo

**Affiliations:** a Biocomputing Unit, Centro Nacional de Biotecnologia (CNB-CSIC), Calle Darwin 3, 28049 Cantoblanco, Madrid, Spain; b Masaryk University, Brno, Czech Republic; c School of Engineering and Applied Science, Yale University, New Haven, CT 06520-829, USA; d Escuela Politecnica Superior, Universidad Autónoma de Madrid, 28049 Cantoblanco, Madrid, Spain

**Keywords:** single-particle analysis, cryo-electron microscopy, parameter estimation, image processing, bias, variance, overfitting, gold standard

## Abstract

Cryo-electron microscopy (cryoEM) has become a well established technique to elucidate the 3D structures of biological macromolecules. Projection images from thousands of macromolecules that are assumed to be structurally identical are combined into a single 3D map representing the Coulomb potential of the macromolecule under study. This article discusses possible caveats along the image-processing path and how to avoid them to obtain a reliable 3D structure. Some of these problems are very well known in the community. These may be referred to as sample-related (such as specimen denaturation at interfaces or non-uniform projection geometry leading to underrepresented projection directions). The rest are related to the algorithms used. While some have been discussed in depth in the literature, such as the use of an incorrect initial volume, others have received much less attention. However, they are fundamental in any data-analysis approach. Chiefly among them, instabilities in estimating many of the key parameters that are required for a correct 3D reconstruction that occur all along the processing workflow are referred to, which may significantly affect the reliability of the whole process. In the field, the term overfitting has been coined to refer to some particular kinds of artifacts. It is argued that overfitting is a statistical bias in key parameter-estimation steps in the 3D reconstruction process, including intrinsic algorithmic bias. It is also shown that common tools (Fourier shell correlation) and strategies (gold standard) that are normally used to detect or prevent overfitting do not fully protect against it. Alternatively, it is proposed that detecting the bias that leads to overfitting is much easier when addressed at the level of parameter estimation, rather than detecting it once the particle images have been combined into a 3D map. Comparing the results from multiple algorithms (or at least, independent executions of the same algorithm) can detect parameter bias. These multiple executions could then be averaged to give a lower variance estimate of the underlying parameters.

## Abstract 2

Single-particle analysis (SPA) by cryo-electron microscopy comprises the estimation of many parameters along its image-processing pipeline. Overfitting observed in SPA is normally due to misestimated parameters, and the only way to identify these is by comparing the estimates of multiple algorithms or, at least, multiple executions of the same algorithm.

## 1. Introduction

Single-particle analysis by cryoEM has become a popular technique to elucidate the 3D structure of biological macromolecules. Thousands of projection images from allegedly the same macromolecule are combined into a single density map that is compatible with the acquired measurements. The signal-to-noise ratio of each of the experimental images ranges from 0.1 to 0.01. This reconstruction process requires the estimation of hundreds of thousands of parameters [the alignment parameters for each experimental image (Sorzano, Marabini *et al.*, 2014 ▸) and whether or not they belong to the structural class being reconstructed]. There are six parameters per image (three Euler angles, two in-plane shifts and one parameter for the class the particle belongs to). Additionally, from a mathematical perspective, the reconstructed volume itself is another set of parameters that must be determined (Scheres, 2012*a* ▸). The existence of many iterative reconstruction algorithms attests to this (Sorzano, Vargas *et al.*, 2017 ▸), although, to a large extent, once the alignment parameters have been fixed there is very little freedom to choose the reconstructed volume. A different perspective is given by Sharon *et al.* (2020 ▸) and all of the previous work by the same group leading to this publication, in which the map is directly reconstructed from the experimental projections without the need to estimate the alignment parameters. This latter family of algorithms is still under development.

For a review of the single-particle analysis technique the reader is referred to Lyumkis (2019 ▸), and for a description of the image-processing pipeline the reader is referred to Sorzano, Jiménez-Moreno *et al.* (2021 ▸).

In this article, we focus on the structural bias; that is, the difference between our estimated structure, 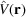 , and the true underlying structure, *V*(**r**). Obviously, we will never have access to the underlying true structure in a single experiment, if only because the measurement noise will cause some random fluctuation around it. We will model our observation as

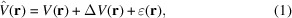

where 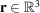 is a spatial location in real space, Δ*V*(**r**) is the structural bias and ɛ(**r**) is a random fluctuation with zero mean. The random noise, ɛ, normally decreases with the number of measurements [for instance, in Unser *et al.* (2005 ▸) we explicitly measured how the 3D reconstruction process attenuated white noise], suggesting that it does not pose a major problem in the current era of the automatic acquisition of thousands of micrographs. The problem is with the bias, Δ*V*, that systematically distorts our structure, preventing us from visualizing the true structure. This bias may be related to missing information, violations of the assumptions of the 3D reconstruction process, incorrect priors about the underlying structure, local minima in the search of the parameters of each image, incorrect use of the programs, software bugs or even the 3D reconstruction workflow itself.

In this article, we have opted for an organization of the work in which all the experiments have been moved to the supporting information. In this way, the main manuscript remains rather narrative and the user is not distracted from the main messages. In Section 2 we set up the statistical framework to analyze bias and variance during the estimation of parameters and to study how they affect the final reconstructed structure. In Sections 3 and 4 we discuss possible experimental and algorithmic sources of bias. In Section 5 we will analyze the currently used tools and recently proposed tools to detect bias. Finally, in Section 6 we draw some conclusions.

## 2. Bias and variance of parameter estimates

Volume overfitting is a feared feature of electron microscopy, and rightly so because it results in incorrect macromolecular structures (see Fig. 1 in Scheres & Chen, 2012 ▸). In the field, it is believed to come from excessive weight on the data, and it is thought to be tackled by providing a suitable weight on a Bayesian prior (Scheres, 2012*a* ▸). Bayesian approaches are handy statistical tools if data are scarce. Interestingly, although the notion of overfitting is generally understood in the structural biology community, to the best of our knowledge there is not a formal, mathematical definition of it in the statistics domain. Overfitting occurs, for example, when the fitted function in a regression problem has too many parameters, so that the function can afford to follow the noise rather than just smoothly following the data trend. Overfitting would be the opposite of the ‘principle of parsimony’ in which a model should have the smallest number of parameters to represent the data adequately. Even this principle is not formally formulated. Instead, statisticians see overfitting as a trade-off between variance and bias of the parameter estimators (Burnham & Anderson, 1998 ▸, chapter 1). Let us assume that we have *x* and *y* observations that are related by a functional relationship plus observation noise, 

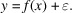

We will perform a regression with a function parametrized by a set of parameters, Θ, such that our prediction of *y* is 

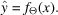

Then, it can be proved that the mean-squared error (MSE) of our prediction is given by (Section 7.3 of Hastie *et al.*, 2001 ▸) 

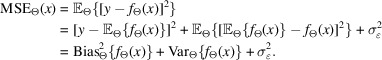

The roles of *y*, *f*_Θ_ and *x* are played by different elements in each one of the problems addressed below, and this formulation should be taken as a generic framework for understanding some of the important properties of parameter estimation.

Probably the simplest model to illustrate this trade-off is regression by *k*-nearest neighbors (kNN). In this technique, the predicted value for a given *x*_0_ is the average of the *y* values of the *k* nearest neighbors of *x*_0_ (for simplicity of notation, let us illustrate the example for univariate predicted and predictor variables, but the same idea could be extended to multiple dimensions). For the kNN regression, the equation above particularizes to (Section 7.3 of Hastie *et al.*, 2001 ▸) 

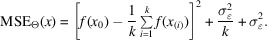

In kNN regression, the complexity of the regression function (its number of parameters) is inversely proportional to *k*. That is, a very large *k* results in very few different predictions (in the limit, eventually all predictions are equal and equal to the input sample mean), with a consequent very low variance of the predictions [Var_Θ_{*f*_Θ_(*x*)}], but a huge bias with respect to the true underlying value *f*(*x*_0_), while a small *k* results in many more output possibilities and therefore better adaptation to the local specificities around *x*_0_ (and consequently low bias), but in a much larger variance of the predictions because the second term is divided by a small *k*.

**Figure 1:**
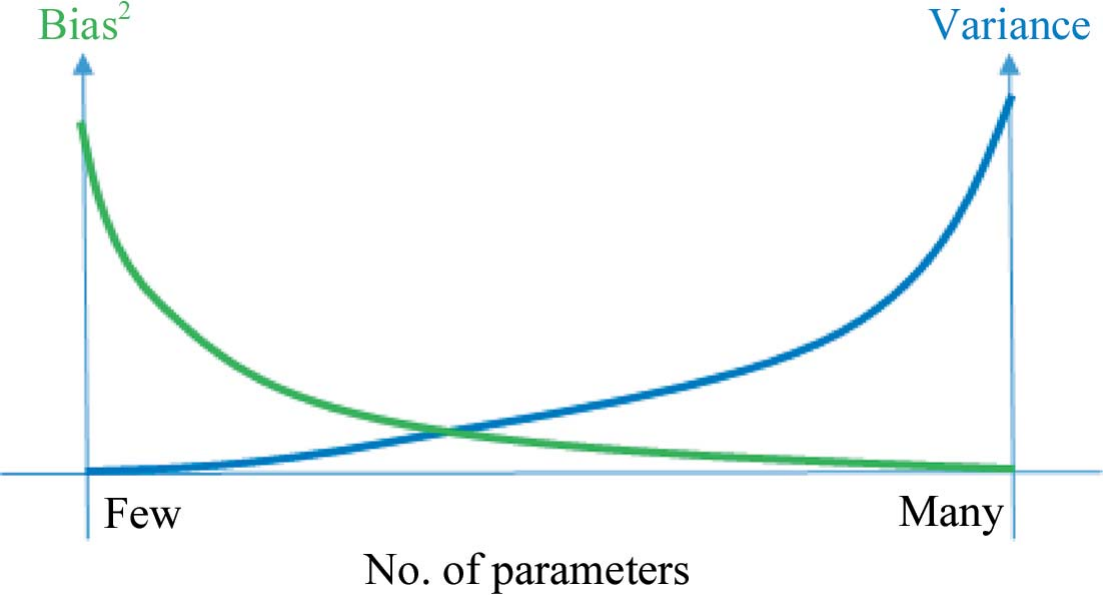
Conceptual trade-off between bias and variance of the parameter estimators depending on the number of parameters.

This example with kNN regression illustrates a much more general principle: as the number of model parameters grows, the bias of the estimated parameters decreases and the variance increases (see Fig. 1 ▸). This is known as the bias–variance trade-off (Section 7.3 of Hastie *et al.*, 2001 ▸). Models with a low number of parameters cannot explain part of the experimental data. In contrast, models with a large number of parameters do explain the data. Still, they have an unnecessarily large variance with respect to a more parsimonious model that explains the data almost equally well. Model-selection methods such as the Akaike’s or Bayesian information criterion (AIC or BIC) try to achieve the minimum of this trade-off between bias and variance (Burnham & Anderson, 1998 ▸). To illustrate this idea, let us analyze the formula of the Bayesian information criterion 

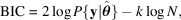

where **y** are the observed data, 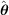 is the set of model parameters, 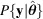 is the likelihood of observing the data given these parameters, *k* is the number of parameters of the model and *N* is the number of observations. The goal is to choose the model that maximizes the BIC. The first term is a data-fidelity term, while the second term is a penalization for the number of parameters in the model.

Although intuitively appealing, having too many parameters is not the explanation for the overfitting observed in EM. Let us consider a set of 100 000 particles of size 200 × 200 pixels. We need to determine a volume of size 200 × 200 × 200 (= 8 000 000 parameters) and 600 000 alignment parameters (five alignment parameters per particle and one additional parameter to decide whether or not the particle belongs to the class that we are reconstructing). This makes a total of 8 600 000 parameters, but there are 4 000 000 000 measurements (pixels). The exact account is not so simple because the quality and the solvability of the map are also related to the angular coverage (Sorzano *et al.*, 2001 ▸; Naydenova & Russo, 2017 ▸; Sorzano, Vargas, Otón, Abishami *et al.*, 2017 ▸; Tan *et al.*, 2017 ▸), which mathematically is also related to the null space of the matrix associated with the set of measurements (Sorzano, Vargas, Otón, de la Rosa Trevín *et al.*, 2017 ▸), but it brings in the idea that the number of measurements largely surpasses the number of unknowns; correlations among pixels are not considered either. Consequently, the reconstruction artifacts observed in the 3D reconstructions do not come from the freedom of the volume to fit the noise (variance), but from a mismatch between the model and the data (data not originating from this model), incorrect estimates of the particle parameters, or goal functions or algorithms that produce biased results. This statement is experimentally supported in this article by multiple experiments addressing different steps along the image-processing pipeline. The estimation of any parameter can easily fall into local minima, calling for robust image-processing algorithms that are capable of reliably estimating all of these parameters in such a noisy environment. In this article, we argue that there are several sources of bias. Some of them are related to the sample while others are related to the algorithm. Among them, the most important at present is the incorrect estimation of the particle parameters.

For example, for the images supposed to correspond to a macromolecule of interest, we must determine whether they all come from a single, homogeneous population of structurally identical particles or exhibit some kind of structural variability. This task is performed by classification of the particles into supposedly homogeneous 3D classes (the cryoEM formulation of this problem is very close to the formulation of mixture models in machine-learning clustering; McLachlan & Basford, 1988 ▸). Misclassifying particles results in 3D reconstructions from a mixture of structurally different objects. For instance, let us imagine that we are trying to obtain the structure of class 1 from *N*_1_ images of that structural class, and we have a mixture with *N*_2_ images of class 2. We will have an estimate of the underlying structure that, in a very simple approximation that assumes linearity of the 3D reconstruction process and the same weight for all images, is 

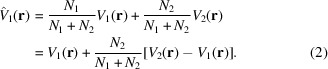

How large the bias that we obtain in our estimate of *V*_1_ is depends on the amount of contamination from class 2, *i.e.* *N*_2_, and the true structural difference between *V*_2_ and *V*_1_. If the 3D reconstruction process is nonlinear (as it is) or the images receive different weights (as they do), then the formula above is not verified in its stated form. However, its simplified version already points out two interesting features that hold in more complicated scenarios: (i) the 3D reconstruction is a mixture of two different structures and (ii) how large our bias in the estimation of *V*_1_ is depends on our number of mistakes, *N*_2_, and the difference between *V*_2_ and *V*_1_. In addition to the difficulties described above, we encounter the additional drawback that the true number of classes is unknown in an experimental setting.

Within a homogeneous population of structurally identical particles, we may encounter the same bias problems if we incorrectly estimate the angular orientation and in-plane shifts of the experimental images or their acquisition parameters (defoci, beam tilt *etc.*). *N*_1_ would now play the role of the particles with correct parameters, *N*_2_ the role of the particles with incorrect parameters and *V*_2_ a 3D reconstruction with the incorrect parameters and their corresponding particles.

One of the major sources of systematic errors when estimating parameters is produced by what we refer to as the ‘attraction problem’, which was rigorously proved in equation (6) in Sorzano *et al.* (2010 ▸). Let us summarize here the main argument. Many algorithms in the field eventually require a comparison between the experimental images and a set of reference images. For example, this comparison is required when assigning an input image to a 2D class, a projection direction or a 3D class. We need to compare the input experimental image with the representative of the 2D class or the reprojection of the current estimate of the map or the 3D class along that direction. The two main tools to perform these comparisons are the Euclidean distance (in real or Fourier space) and the correlation (actually, using relatively mild hypotheses, it can be proved that the reference that maximizes the correlation is the same as the one that minimizes the Euclidean distance). As soon as one of the references starts to get more images, its background will be less noisy because of the higher averaging caused by the larger number of images. However, in the correlation or Euclidean distance calculation the background also contributes, and that with lower noise will contribute less to the Euclidean distance. In this way, it will seem closer to some experimental images, even if these do not correspond to the signal represented by the reference. Consequently, more images are assigned to that reference, and the larger averaging effect is positively reinforced. Euclidean distance and correlation can be considered ‘classical’ image-similarity tools. With the advent of deep learning, a new approach that has not yet been adopted in cryoEM is the learning of the distance function itself (Wang *et al.*, 2014 ▸). This possibility could open new research routes towards more robust image classification and alignment.

Overall, in current practice, we would say that incorrect parameter estimation is the major source of bias in cryoEM. Throughout this article, we discuss several strategies to identify and try to prevent it. One of the most powerful strategies is to analyze the parameter estimates using several independent algorithms, or at least multiple runs of the same algorithm with random initialization. This is in line with the current trend in machine learning of using ensemble approaches such as boosting (an ensemble of many high-bias models that decreases the overall model bias and variance) and bagging (sub­sampling data with replacement decreasing the overall model variance) (Hastie *et al.*, 2001 ▸).

## 3. Experimental sources of bias

Several sources of bias are primarily related to the sample itself rather than the method of processing the data set, although everything is inter-related. In the following we discuss some algorithmic solutions that we can adopt to prevent structural bias due to these problems.

### 3.1. Use of incorrect particles

The 3D reconstruction algorithm will assume that a single structure is perfectly compatible with the measurements except for random, zero-mean noise that is superposed on the projection images. This assumption is violated by protein denaturation, conformational heterogeneity and the presence of contaminants, aggregations, disassembled particles, radiation damage, particle superposition within the ice layer *etc.* In a way, deciding whether a patch of a micrograph is a particle or not is the first parameter that we must determine.

All particle pickers have false positives (objects incorrectly identified as particles) and false negatives (missed particles). At present, the trend is to set the picking parameters so that ‘all’ particles are selected. The idea is to maximally exploit the structural information present in the micrographs. However, this puts pressure on the 2D and 3D image processing to identify particles that do not really correspond to isolated, single particles of the macromolecule under study. Despite the solid image-processing and artificial intelligence background of the most widely used pickers nowadays (Abrishami *et al.*, 2013 ▸; Bepler *et al.*, 2019 ▸; Wagner *et al.*, 2019 ▸), all of them have a false-positive rate that ranges between 10% and 30% depending on the data set (Sanchez-Garcia *et al.*, 2020 ▸). Algorithms such as *Deep Consensus* (Sanchez-Garcia *et al.*, 2018 ▸) were specifically designed to take all of these candidates to particle centers and apply a deep-learning algorithm to learn their commonalities and decide which of the coordinates really correspond to a particle and which are false positives. There are also algorithms that try to remove the coordinates of protein aggregations, carbon edges, contaminants or any other sample defect (Sanchez-Garcia *et al.*, 2020 ▸). In our experience, the combination of *crYOLO* (Wagner *et al.*, 2019 ▸), *Xmipp* picking (Abrishami *et al.*, 2013 ▸), *Deep Consensus* (Sanchez-Garcia *et al.*, 2018 ▸) and *Micrograph Cleaner* (Sanchez-Garcia *et al.*, 2020 ▸) produces very few false-positive particles. Any denoising algorithm such as that described by Bepler *et al.* (2020 ▸) can also help to produce cleaner micrographs in which particle finding is simplified and, consequently, presumably more accurate. A dangerous practice is to select particles using a reference external to the study, because it may lead to biased reconstruction, as in the famous case of the HIV trimer (Mao, Wang *et al.*, 2013 ▸; Mao, Castillo-Menendez *et al.*, 2013 ▸; Henderson, 2013 ▸; Subramaniam, 2013 ▸; van Heel, 2013 ▸).

Obviously, an important consequence of not using the correct particles for the macromolecule being reconstructed is that the presence of incorrect particles will contaminate the 3D reconstruction, as described in equation (2).

As we mentioned above, the 3D reconstruction process assumes that the projection of the reconstructed map matches the experimental projection except for the noise. This assumption is violated in a series of cases.

(i) If our particles are not isolated (they can be nearby or superposing; Noble *et al.*, 2018 ▸), they do not correspond to the structure under study (but instead to contaminants, images of ice, aggregations, particles on carbon *etc.*) or they correspond to the structure being reconstructed plus some attached flexible matter (antibodies, surrounding membrane, factors that may or may not be bound *etc.*). In these circumstances, the 3D alignment algorithm will try to satisfy the reconstructed particle and its surrounding matter. As shown in Supplementary Experiment 1, the current practice of taking as many particles as possible, disregarding their quality and hoping that the algorithm will manage is very likely to be counterproductive. The reader may note that the induced artifacts are not constrained to the area outside the macromolecule. Inside the macromolecule there are also important structural differences caused by the nearby entities.The solution to this problem would consist of powerful particle picking, as described above, and 2D image analysis: particle screening (Vargas *et al.*, 2013 ▸), 2D classification (Sorzano *et al.*, 2010 ▸; Scheres, 2012*b* ▸; Punjani, Rubinstein *et al.*, 2017 ▸) and outlier analysis of the classes (Sorzano, Vargas *et al.*, 2014 ▸). Choosing those particles from classes in which particles are isolated should be preferred, and if this is not feasible due to the high concentration of particles, then making the 3D alignment with a tight mask (used for alignment, not reconstruction) could help, but in general this is not a solution. Note that the tight mask can be used in two places: (i) to construct the reference mask to apply to the input volume so that it removes the information around the particles and (ii) to mask the reconstructed map so that we can ‘hide’ the artifacts outside the particle, but not the structural modifications inside the mask. We do not object to its first use, as removing artifacts from the reference volume prevents artifactual features from acting as noise anchors. However, we discourage its second use as we may not see possible biases whose effect is more easily detected outside the macromolecule.As a final warning, we should be aware that choosing particles only from ‘good-looking’ 2D classes does not guarantee good particles due to the attraction problem in 2D classes (Sorzano *et al.*, 2010 ▸) (depending on the algortihm used, for example *RELION* 2D or *cryoSPARC* 2D) or the scattering of bad particles into the existing classes (Sorzano, Vargas *et al.*, 2014 ▸).
(ii) If particles are misclassified during the 3D classification (either because the classification is performed attending to the angular orientation of the particles, several populations are mixed or because differences in the 3D classes are found by incorrect angular assignments; Sorzano *et al.*, 2020 ▸). Supplementary Experiment 2 shows examples of the instability of the 3D classification process and 3D attraction problems.A possible strategy to avoid misclassification could be to repeat the 3D classification process several times and with different algorithms (Scheres *et al.*, 2007 ▸; Scheres, 2012*a* ▸; Lyumkis *et al.*, 2013 ▸; Punjani, Brubaker *et al.*, 2017 ▸), keeping only those images that are consistently classified together. The reason is that performing this classification is extremely challenging for currently existing algorithms, and we cannot just take ‘the first classification result’, as it will most likely contain important mixtures of different subpopulations.

### 3.2. Use of incorrect symmetry

If our structure is pseudosymmetric and we reconstruct it as symmetric, we will lose the small differences between subunits. If our structure has some symmetry parameters, such as a helix, and we use different parameters, we will strongly distort our structure. These symmetry-related biases can occur in standard single-particle studies (Ludtke *et al.*, 2004 ▸), electron crystallography (Gil *et al.*, 2006 ▸; Biyani *et al.*, 2018 ▸), helical reconstructions (Egelman, 2014 ▸) and studies of icosahedral viruses (Koning *et al.*, 2016 ▸). Additionally, macromolecules are intrinsically flexible objects that could be fluctuating around an energetically stable solution (Sorzano *et al.*, 2019 ▸), and these fluctuations automatically break all symmetries at high resolution.

Pseudosymmetry is currently one of the most active lines of research. This is useful for analyzing macromolecules with almost equivalent subunits and for analyzing the asymmetric part of particles in which a part is symmetric or has a different symmetry (for instance, nucleic acids inside an icosahedral virus capsid, virus portals *etc.*). One of the solutions is to perform symmetric and asymmetric reconstructions to verify the consistency between the two structures. However, this option is not always feasible due to the 3D attraction problem. Alternatives are symmetry expansion (Scheres, 2016 ▸) or symmetry relaxation (Huiskonen, 2018 ▸), in which the method tries to separate the particles into structurally homogeneous groups. Another solution would be to analyze the set of images around its symmetric conformation using continuous heterogeneity tools (Dashti *et al.*, 2014 ▸; Jin *et al.*, 2014 ▸; Haselbach *et al.*, 2018 ▸) and focusing on groups of particles according to their deformation parameters (Jin *et al.*, 2014 ▸).

### 3.3. Missing information

If we lack information from some projection directions, this may cause, depending on which directions are missing, empty regions in the Fourier domain for which we simply do not know what the protein looks like. Filling this region with zeroes is usually a bad choice as it results in an elongation of the structure along the missing direction. The absence of measurements in some regions of the Fourier domain is well known in the field because it occurs in some data-collection schemes such as random conical tilt (Radermacher & Hoppe, 1980 ▸; Radermacher *et al.*, 1987 ▸; Radermacher, 1988 ▸; Sorzano, Alcorlo *et al.*, 2015 ▸) and orthogonal tilt (Leschziner & Nogales, 2006 ▸). An ideal single-particle analysis should not suffer from this problem as in principle particles can be acquired from any possible orientation. An alternative to filling the Fourier space with zeroes is to provide information that guarantees some kind of continuity (Moebel & Kervrann, 2020 ▸). However, many projects in SPA face the problem of uneven angular distributions, potentially causing severe artifacts in the 3D reconstructions. Some of these uneven angular distributions are truly caused by a preferential interaction of the macromolecule with the water–air interface or the sample support (Tan *et al.*, 2017 ▸; Noble *et al.*, 2018 ▸). In these cases, the lack of experimental data could be complemented with *a priori* volumetric constraints (external surface, total mass, non-negativity *etc.*; Sorzano *et al.*, 2008 ▸). This is not an easy task as it involves iterative reconstruction algorithms, which are now in disuse because they are much slower than their Fourier gridding counterparts. At present, it is preferred to tilt the sample (Tan *et al.*, 2017 ▸) or to look for different sample-preparation conditions that do not cause preferential views.

## 4. Algorithmic sources of bias

Structural biologists are very much aware of the problems referred to above and try their best to overcome them. However, algorithmic reasons may also prevent us from achieving an unbiased estimate of the structure under study. Some of them are very well known, such as the dependence of the final structure on the initial guess or the existence of software bugs. Some others are suspected, such as the existence of local minima in the parameters to estimate. Yet others are buried deep in the 3D reconstruction and classification process and are seldom exposed, but are critical.

In this section, we discuss sources of bias that are more related to the image processing itself. We focus on problems that presently remain a bottleneck or that have received less attention from the community. The initial volume problem is of primary importance. As such, it has received all kinds of attention, from descriptions of the problem to algorithmic proposals to tackle it. Although it can cause really poor results if it is not properly selected, our view is that it is now no longer a major bottleneck in most projects as one of the many existing algorithms will be able to find a suitable initial volume. However, our view is that at present the use of incorrect parameters for the particles is the greatest source of structural bias (along with the population mixture that is still observed after 3D classification). The 3D attraction problem causes a major algorithmic problem in some experiments in which the angular assignment is totally biased. Incorrect masking can be a source of structural bias if it truncates part of the structure or leaves extra masses that do not correspond to the macromolecule under study. Still, otherwise, it is not a large challenge except in that it may give us a false sense of good quality by inflating the Fourier shell correlation (FSC). Finally, the image metric and 3D reconstruction algorithm are sources of bias that have never been put forward. Although they do not represent a major problem, it is worth enumerating them in this article and making users aware that the choice of the 3D reconstruction algorithm also introduces its own contribution to the reconstructed structure that might be confounded with true structural features of the macromolecule being reconstructed.

We may identify the following sources of bias induced during the image-processing procedure.

### 4.1. Initial volume

The dependence of the final structure on the initial volume used to be one of the most severe problems some years ago (Henderson, 2013 ▸; Subramaniam, 2013 ▸; van Heel, 2013 ▸). The 3D alignment and reconstruction process is normally some variant of gradient descent. For this reason, the starting point of the iterations plays a crucial role in the optimization process. This is the reason behind the well known Einstein from noise effect (Shatsky *et al.*, 2009 ▸). Several solutions have been proposed in recent years to tackle this problem, such as stochastic optimization algorithms (Ogura & Sato, 2006 ▸; Elmlund *et al.*, 2013 ▸; Vargas *et al.*, 2014 ▸; Punjani, Brubaker *et al.*, 2017 ▸), slowly converging algorithms (Scheres, 2012*a* ▸; Sorzano, Vargas *et al.*, 2015 ▸) and consensus algorithms (Sorzano, Vargas *et al.*, 2018 ▸; Gómez-Blanco *et al.*, 2019 ▸). Thanks to all of these new algorithms, the initial volume dependence is no longer a major bottleneck in the image-processing pipeline as long as these algorithms are judiciously used. There are also ways to validate the initial volume through external measurements such as SAXS data (Jiménez *et al.*, 2019 ▸). In any case, it should be noted that a bad choice of the initial volume very often leads to erroneous results.

### 4.2. Incorrect alignment parameters

One of the most important sources of bias is an inaccurate estimation of the alignment parameters. Stewart & Grigorieff (2004 ▸) reported important differences in the image alignment depending on the goal function that is being optimized. Consequently, differences in the angular assignment between different programs should be expected. We can think of two different kinds of errors: (1) the alignment parameters found are a small, randomly perturbed version of the true (although unknown) alignment and (2) the alignment parameters found are in a region of the projection sphere or in-plane alignment totally unrelated to the true alignment. In any case, both kinds of mistakes result in an error in the particle orientation, with some errors larger than others depending on whether that specific particle is in case (1) or (2) (see equation 2).

In a previous section, we discussed projects with incomplete angular coverage due to experimental reasons. It is less well known that the angular assignment algorithm itself can also cause this bias in the angular assignment through the previously mentioned 3D angular attraction (Sorzano, Semchonok *et al.*, 2021 ▸). As shown in Supplementary Experiments 2 and 3, the 3D attraction effect is a problem that severely affects the validity of the reconstructed map. This behavior has a doubly deleterious effect: firstly, it places experimental projections in incorrect directions, causing structural bias by a mixture of signals and, secondly, it may deplete low-populated directions in favor of nearby directions, causing structural bias by lack of information.

Supplementary Experiments 4 and 5 show that, depending on the data set, uncertainty about the 3D orientation and in-plane alignment of experimental images affects 10–50% of the data set (note that these numbers are based on our experiments and different data sets may yield different limits). Two different algorithms may disagree in the angular assignment of up to 50% of the images (Supplementary Experiment 4). This disagreement may also be found in multiple runs of the same algorithm (Supplementary Experiment 5). This indicates the variability of the alignment parameters, but also that for any particular execution a fraction of the parameters are significantly biased. These inconsistent parameters can be identified if the outputs of several program runs are compared, but this is seldom performed. The extent of the effect in a particular study is impossible to determine if only one 3D classification or angular assignment is performed for a particular set of images. Its detection necessarily requires multiple independent estimations of the underlying parameters (class membership and/or angular assignment), preferably using algorithms based on different mathematical principles. After comparing their different outputs, one may identify those particles for which the estimates agree. Unfortunately, for those for which they disagree it is difficult at the moment to decide which are the true parameters. Some algorithms are more prone to 3D attraction. Those related to a Euclidean distance between two images (such as *RELION*, *cryoSPARC* and *cisTEM*) are more susceptible to suffering it [see equation (6) of Sorzano *et al.* (2010 ▸) for the mathematical explanation]. *Xmipp HighRes* uses a weighting scheme based on the significance of two score functions in its global alignment stage, which might be the reason for its higher immunity to this problem. It should be noted that an absolute consensus algorithm that only keeps the images for which all alignment algorithms agree on their angular assignment would not solve the 3D attraction problem, as the ‘attracted’ algorithm would prevent the rest from filling the depleted regions. More creative strategies, probably involving three or more independent assignments, should be devised in this case, and this problem is foreseen to be an active research topic in the future.

Another way to identify misaligned particles is through the use of multiple objective functions. Most algorithms optimize a single objective function (log likelihood in the case of *RELION* autorefine or cross-correlation in a maximal circle in *Xmipp HighRes* local alignment). From the point of view of a single numerical observer, it is normally not possible to recognize the presence of misaligned particles. However, the calculation of several similarity measures may help recognize the set of misaligned particles or nonparticles still in the data set. Supplementary Experiment 6 shows how the calculation of the *Xmipp HighRes* local alignment similarity measure can identify two subpopulations where *RELION* autorefine cannot. In general, each different similarity measure ‘sees’ different features of the same alignment. Using tools such as the different similarity measures shown above or the alignability of the particles shown in Vargas *et al.* (2016 ▸, 2017 ▸) and Méndez *et al.* (2021 ▸), we should also be able to identify those particles for which the angular assignment is in doubt. We have also found it very useful to perform a 3D classification of the particles in two classes without re-estimating the angles. Particles with an incorrect alignment tend to cluster in one of the classes, while the other class retains the particles with good alignment (Sorzano *et al.*, 2020 ▸).

A similar situation of alignment bias occurs if the handedness of the images is mixed, as reported in Sanz-García *et al.* (2010 ▸) (see Supplementary Experiment 7). Once the angular assignment falls into this situation, it is challenging to disentangle the hand mixture. A possible way is by constructing an initial volume from the particles assigned to a 3D class and verifying that the reconstructed structure resembles it.

### 4.3. Incorrect CTF correction

Another source of bias may come from inaccuracy in the estimation of the CTF parameters. In its most simplified version, the CTF formula is sin(πλΔ*fR* ^2^ + …), where λ is the electron wavelength, Δ*f* is the defocus and *R* is the frequency at which we evaluate the CTF (Sorzano *et al.*, 2007 ▸). The two most important parameters of the CTF are the microscope voltage (which determines the electron wavelength) and the micrograph defoci (Sorzano *et al.*, 2009 ▸). Zhang & Zhou (2011 ▸) stated that the maximum defocus error to achieve high resolution should be below 100 Å. Larger errors would result in incorrect compensation of the phase shift introduced by the microscope. In the CTF challenge, the discrepancy between different CTF estimation software programs was around 200 and 300 Å (Marabini *et al.*, 2015 ▸). As with any other parameter, random fluctuations around the true value must be expected, and these will naturally limit the maximum achievable resolution. However, if these estimation errors are not random (as assumed, for instance, in Penczek *et al.*, 2014 ▸) but systematic, we may consistently overcompensate or undercompensate some frequencies (this effect is significant at medium frequencies; at high frequencies the CTF oscillates more rapidly and it is more difficult to make systematic errors). Systematic errors in the CTF normally translate into a dark halo around the macromolecule, as seen in many EMDB entries (see, for instance, EMDB entries EMD-20310 and EMD-20702 as examples of recent releases from October 2019), and a haze on top of the macromolecule. In Supplementary Experiment 8 we show an example in which the dark halo around the particle and the haze on top of it are generated by systematic errors in the estimation of the CTF defocus. Note that in the CTF formula the pixel size participates through the frequency term [*R* = *i*/(*NT*_s_), where *i* is the index of the frequency term in the fast Fourier transform of the input image, *N* is the image size, which is assumed to be square for simplicity, and *T*_s_ is the pixel size or sampling rate]. Consequently, a small error in the pixel size also systematically causes miscorrections of the phase flip. In Supplementary Experiment 8 we also show how systematic errors in the pixel size also translate into dark halos. At present, it is customary to refine the CTF parameters per particle locally (Zhang, 2016 ▸; Bartesaghi *et al.*, 2018 ▸; Sorzano, Vargas *et al.*, 2018 ▸; Zivanov *et al.*, 2018 ▸). Although these optimizations are not expected to be particularly biased, the amount of signal available to estimate the CTF parameters per particle is so small that large variances should be anticipated. To the best of our knowledge, there has not been any rigorous work that has tried to estimate the variability of the per-particle parameters. In real practice, dark halos around the reconstructed maps are very often observed. These are probably caused by a mixture of random and systematic errors in the pixel size (which should be small and can be corrected with a recalibration using an atomic model of the structure) and random and systematic errors in the defocus estimates (which can be minimized by averaging the defocus values reported by several CTF estimation algorithms). The problem is not the halos and hazes themselves, which the isosurface visualization programs can easily ignore. The problem is that we know that the presence of the halo and haze implies the existence of fine structure differences inside the macromolecule, as shown in Supplementary Experiment 8. Note that we cannot show evidence that there are systematic errors in determining the defocus in published experiments. However, we can reproduce the same kind of errors as those in published experiments by forcing a systematic error in the defocus determination.

### 4.4. Image normalization

The 3D reconstruction process assumes that the acquired images are projections of the macromolecule under study in different poses. The weak phase object approximation gives the relationship between the projection image and the volume to be reconstructed (Koeck & Karshikoff, 2015 ▸). In this approximation, a transmitted beam gives rise to a baseline rate of electron arrivals modulated by the matter along their path (the weak phase object approximation states that the modulation is linear). However, this model implies that the raw images acquired by the microscope must be normalized before entering the 3D reconstruction process (for example the ice thickness is not the same for all particles). The normalization normally sets the statistical properties of the ice to some prespecified values (Sorzano *et al.*, 2004 ▸). However, this normalization is affected by outlying pixels, nearby particles, contaminations or carbon edges, illumination gradients, inhomogeneous camera gain images (Sorzano, Fernández-Giménez *et al.*, 2018 ▸) *etc*. For this reason, the particle normalization must be refined in order to make the projection images maximally consistent with the reconstructed volume (Scheres *et al.*, 2009 ▸; Sorzano, Vargas *et al.*, 2018 ▸). We may think of the normalization process as a linear transformation of the input images *I*′ = *aI* + *b*. Systematic errors in *b* translate into a sphere of density around the reconstructed molecule (the reason is that the backprojection of an additive constant in all possible projection directions is not a constant map, but a sphere whose density increases with the radius up to the maximum radius that can be embedded in a box of the size of the particles). This kind of systematic error is seldom seen in 3D reconstructions of single particles. Instead, random errors in *b* should be more common. Similarly, it is hard to think up situations in which the image-normalization process systematically biases *a*. However, images participate in the 3D reconstruction process with some weight (Grigorieff, 2007 ▸; Scheres *et al.*, 2007 ▸; Sorzano, Vargas *et al.*, 2018 ▸), and one can imagine systematically high or low weights depending on the projection direction (for instance, the attraction problem in 3D places more images along specific directions, resulting in a higher weight of that direction with respect to the rest). This situation could not be distinguished from systematic errors in *a*.

### 4.5. Incorrect masking in real or Fourier space

Incorrect masking in real space or Fourier space can either cut out valid regions of the map or, on the contrary, leave regions that do not correspond to the structure of interest but may serve as anchors for noise alignment (a related problem can be seen for membrane proteins, where the density of the membrane may drive the angular alignment in undesired ways).

The use of masks during alignment is recommended. They prevent the alignment from being driven by artifacts around the 3D reconstruction that are unrelated to the structure under study (Sorzano, Vargas *et al.*, 2018 ▸; see also Supplementary Fig. 2). The same logic applies to masks in the Fourier domain: the FSC can serve as an indicator of the reliability of the different Fourier components. This reliability can be explicitly used during the alignment phase to limit the amount of unreliable content that can serve as noise anchors (Scheres, 2012*a* ▸; Grant *et al.*, 2018 ▸; Sorzano, Vargas *et al.*, 2018 ▸).

We should distinguish between real-space and Fourier space masks for angular alignment or as post-processing tools. The use of real-space masks after reconstruction should be discouraged because they could hide possible biases. Similarly, modifications of the amplitude spectrum after reconstruction, such as the *B*-factor correction, normally lead to a biased overboosting of the high-frequency components (Ramírez-Aportela *et al.*, 2020 ▸), resulting in publicly deposited maps that do not comply with the expected behavior of the diffraction of macromolecules (Vilas, Vargas *et al.*, 2020 ▸). Other modifications that try to match the amplitude spectrum of the reconstructed map to that of its atomic model (Jakobi *et al.*, 2017 ▸) explicitly address the minimization of this bias as long as the atomic model is correct (otherwise, this match would induce another bias). Interestingly, current post-processing approaches such as that in *RELION* basically amount to a mask and *B*-factor correction. After this transformation, the FSC significantly improves, reporting a higher resolution for the reconstructed map (see Supplementary Experiment 9). However, this increase in resolution is merely due to the change of the mask between that used during reconstruction and that used during post-processing because the FSC is invariant to radially symmetric transformations such as the *B*-factor correction (Sorzano, Vargas, Otón, Abrisham *et al.*, 2017 ▸). Masking in real space translates into a convolution in Fourier space [we denote the Fourier transforms of the estimated map and the applied mask 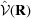 and 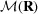 , respectively], 

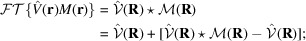

that is, we are biasing our reconstructed volume by another volume whose Fourier transform is 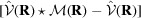 . The absence of a mask is equivalent to a constant mask of value 1 everywhere. Its Fourier transform would be a delta function in Fourier space, and the bias term would be equal to zero. However, tight masks are significantly broad in the Fourier domain, resulting in a large bias, and as shown in Section 5.1 this can make the FSC arbitrarily large, as shown in Supplementary Experiment 9. It should also be noted that a bias with respect to the reconstructed map is not necessarily bad, as the bias should be measured with respect to the true underlying structure, not the reconstructed map. In this regard, the masked volume may be closer to the underlying structure than the reconstructed map if the mask removes map artifacts. Unfortunately, the true structure is never known, and introduction of the mask and its effect on the FSC may result in overconfidence in the quality of the map.

### 4.6. The 3D reconstruction algorithm

As we have seen, some metrics may be better suited than others to identify population mixtures or misaligned particles. These metrics are translated into different weights of the particles in the 3D reconstruction. In turn, this projection-weighting scheme plays an important role in the final reconstruction. For instance, incorrectly aligned particles would not have any effect if their weight is minimal. On the other hand, the possibility of assigning multiple weights to the same image in different orientations will necessarily introduce structural bias in the 3D reconstruction, especially if these weights are similar.

Strictly speaking, the voxel values of the reconstructed map are parameters to determine (8 000 000 in our example in Section 2). Still, once the alignment parameters have correctly been determined (600 000 in the example in Section 2), an unbiased determination of the volume parameters is almost guaranteed with the existing algorithms (Penczek *et al.*, 2004 ▸; Scheres, 2012*b* ▸; Abrishami *et al.*, 2015 ▸; Sorzano, Vargas, Otón, de la Rosa-Trevín *et al.*, 2017 ▸). In this way, most of the effort should be concentrated on determination of the alignment parameters. In the EM community, these parameters have been considered to be nuisance (secondary) parameters (Scheres, 2012*a* ▸; Lyumkis *et al.*, 2013 ▸; Punjani, Brubaker *et al.*, 2017 ▸) arising from some statistical distribution with a Gaussian or uniform prior. This assumption has proved to be very useful in converging from a wide range of initial volumes, as shown by the success of these methods. However, a consequence of adopting a maximum-likelihood approach is that an image is allowed to occupy multiple orientations with different probabilities. This is a violation of the image-formation model, as an image truly arises from only one, although unknown, orientation. Projection matching does not suffer from this drawback (although it has others, such as a much smaller radius of convergence). An image can have a single set of alignment parameters. As argued in Sorzano, Vargas *et al.* (2018 ▸), the probability of making an angular error if a single projection direction is allowed is lower than that of making an angular error if two or more projection directions are allowed (because all except at most one must necessarily be wrong). In Supplementary Experiment 6 we show the distribution of the number of significantly different alignment parameters contributing to each of the experimental images in *RELION*. As can be seen, most of the images have between 1 and 10 significant contributions, with a maximum of about 200. This multiplicity of orientations is translated into a weighting scheme that places the same image at different orientations with different weights. In Supplementary Experiment 10, we show the difference between the 3D reconstruction performed within *RELION* autorefine and *RELION* reconstruction with the same angular distribution. The weighting scheme in *RELION* autorefine results in a low-pass filter of the reconstructed map and a low-frequency white halo superposed on the map. These differences with respect to the true underlying structure are a different kind of bias, in this case caused by the weighting scheme of the reconstruction algorithm.

Maximum-likelihood methods were extended to Bayesian methods by adding a prior on the Fourier coefficients of the reconstructed map (Fourier components that are independent of each other and whose real and imaginary parts are also independent). This prior acts as a low-pass filter (Scheres, 2012*a* ▸) and, as for any prior, it results in a regularization that unavoidably leads to bias (Fessler, 1996 ▸; this is the very purpose of Bayesian methods when data are scarce). Additionally, the specific prior used in the community so far has experimentally been shown to be incorrect for macromolecular structures (Sorzano, Vargas, Otón *et al.*, 2015 ▸), and consequently as an incorrect prior it systematically biases the reconstructions obtained with this objective function. Still, this prior has proved to be very useful for its EM application, although in the future research on new priors based on the nature of macromolecules could be exploited. These priors would not bias the reconstruction process as they would incorporate prior knowledge matching with the objects being imaged.

Overall, the problem of obtaining an incorrect structure is an open problem in the field. How incorrect it is depends on the countermeasures that we have taken to prevent structural bias. Heymann *et al.* (2018 ▸) reached a similar conclusion from the outcome of the map challenge and suggested a set of safe practices that are very much in line with those suggested here. In the following section, we show that our most common strategies to prevent (gold-standard data analysis) and detect (Fourier shell correlation) biased reconstructions can be fooled by systematic errors so that additional measures are necessary.

## 5. Detection and avoidance of bias

In this section, we discuss the tools that are currently in use to detect biased reconstructions. As explained, it is easier to detect biased estimates of the different parameters than their combined effect in the reconstructed map. We start by analyzing the FSC as the most widely used map-quality tool. We show that this tool can easily be fooled by systematic errors (the kind of errors that bias gives rise to). Similarly, it is easier to avoid bias in the various parameter-estimation steps than when splitting the data into two halves from the beginning. We argue that this practice is not common among data-analysis applications as it underutilizes the available data and cannot guarantee the lack of bias. Additionally, we show that splitting the data into two is not necessary to avoid bias. Practices closer to cross-validation or separation into training and test data sets could instead be adopted. Finally, we discuss the current implementation of phase randomization. The original idea is worth pursuing, but its current implementation does not adhere to the original plan.

### 5.1. On the use of the FSC to detect overfitting

The most common tool to detect the overfitting is the FSC between the two maps, 

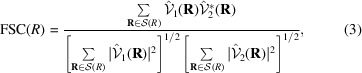

where **R** is the 3D frequency vector, *R* is its magnitude, 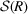 is the Fourier shell whose center has radius *R*, and 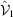 and 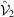 are the Fourier transforms of the two maps reconstructed from the two data halves. [Note that in this formulation we are not analyzing the statistical distribution of the FSC, and this is why we have not further expanded 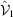 and 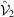 into their deterministic and random components. For a deep analysis of these distributional properties, the reader is referred to Sorzano, Vargas, Otón, Abrishami *et al.* (2017 ▸).] If both reconstructions are biased, *V*_1_ + Δ*V*_1_ and *V*_2_ + Δ*V*_2_, and the FSC becomes 

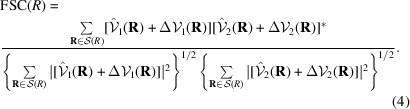

With this measurement, the FSC can be made arbitrarily close to 1 by making 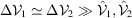 . This bias of the FSC is at the core of some of its reported failures (Borgnia *et al.*, 2004 ▸; Egelman, 2014 ▸; Subramaniam *et al.*, 2016 ▸; Tan *et al.*, 2017 ▸).

We may pose the 3D reconstruction problem as that of estimating a set of parameters **Θ** that include the reconstructed volume, the 3D alignment parameters of each of the experimental projections and any per-particle imaging parameter. The problem is estimating **Θ** from the observed data **y**, 

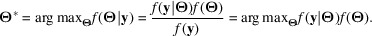

This is a Bayesian regression problem, as stated in Scheres (2012*a* ▸). The first term aims to look for a solution that is consistent with the acquired data. The second term looks for a solution that is consistent with what is known, in general, about biological macromolecules. The drawback of the Bayesian approach is that, for the moment, we do not have a realistic prior for the set of macromolecules being reconstructed. In any case, as with any other regression problem in statistics, the validity of the result should be include the residuals of the regression; that is, comparing the observed **y** with the predicted 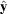 . Different strategies, such as regressing with a large subset of the data and evaluating with a small subset, could be devised (Ortiz *et al.*, 2019 ▸), as is the standard practice in statistics and X-ray crystallography (free *R* value; Brünger, 1992 ▸). This is also the spirit of measures based on the spectral signal-to-noise ratio (SSNR; Penczek, 2002 ▸; Unser *et al.*, 2005 ▸). However, it is not at the core of the FSC. The FSC compares two sets of regression parameters 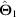 and 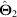 . This has the drawback of being heavily affected by bias: systematic errors are rewarded by the FSC. There is a connection between the FSC and the SSNR when the errors are supposed to be random. However, the functional nature of the relationship is unknown (Sorzano, Vargas, Otón, Abrishami *et al.*, 2017 ▸), and its sensitivity to bias should be considered before adopting it as a universal descriptor of the map quality.

At this point, we would like to highlight that the 0.143 threshold normally used in the field is derived under the assumption of no bias and linearity of the 3D reconstruction process. This latter assumption is broken by some algorithms, for example *Xmipp HighRes* (Sorzano, Vargas *et al.*, 2018 ▸).

As a consequence, the FSC between two halves for these algorithms may sometimes not cross the 0.143 threshold (in Supplementary Experiment 10, we show the impact of the nonlinear processing of *Xmipp HighRes* on the FSC). For this class of algorithms, we have heuristically found that a threshold of 0.5 is often a better estimate of the resolution (despite this concept being ill-defined). This tends to be true not only for *Xmipp HighRes* but in general for most 3D reconstruction algorithms that we have used (*RELION*, *cryoSPARC* and *Xmipp*). We have normally observed that the FSC = 0.143 resolution, in most reconstruction algorithms, is usually the best resolution in the best voxel of the local resolution map (Vilas *et al.*, 2018 ▸; Ramírez-Aportela *et al.*, 2019 ▸; Vilas, Tagare *et al.*, 2020 ▸) and that the FSC = 0.5 resolution is more representative of the most common local resolution value.

We have also observed that the FSC typically presents a change of decaying regime at a frequency that is better related to the frequency at which the map is reconstructed (Supplementary Experiment 12). However, it is difficult to determine these regime changes automatically, and a straightforward, objective criterion cannot be given at this moment.

### 5.2. The gold standard and cross-validation

Throughout this article, we have analyzed the most common sources of bias in cryoEM single-particle analysis. For each of the different sources, we have suggested ways to detect and avoid these biases. The most common way to avoid overfitting in cryoEM is the so-called gold-standard data processing, which divides the data into two independently processed halves (Scheres & Chen, 2012 ▸). The idea is based on previous work (Grigorieff, 2000 ▸) and, as stated in Chen, McMullan *et al.* (2013 ▸),

Grigorieff showed that when signal-to-noise ratio in the images becomes low enough, it is impossible to avoid overfitting when the two half sets are aligned against the same reference structure, regardless of how the procedure is initiated. He was the first to conclude that a reliable estimation of resolution using FSC can be obtained only when the two half datasets are independently aligned against two independent reference structures.

However, a careful reading of Grigorieff (2000 ▸) reveals that what he showed was an experiment in which, with low SNR and a low number of images, the FSC of two halves aligned against the same reference was not representative of the FSC of the full set against the known ground truth. From this experiment, we cannot take as a general result that the data set needs to be split into two halves aligned to independent references. One of the main differences from this result, 20 years later, is that the number of particles used nowadays largely outpaces the number of particles with which this experiment was performed (1000 images).

Actually, this procedure of splitting the data into two halves goes against the most advanced practices in statistics. The standard recommended approach would be cross-validation (dividing the data set into *K* pieces, typically *K* = 10, processing *K* − 1 to produce the map and using the remaining piece to evaluate the quality of the map; the process is repeated *K* times, with each one of the pieces playing the role of the validation subset; Picard & Cook, 1984 ▸). This approach is computationally expensive since the full 3D alignment and reconstruction process must be repeated *K* times, and it may have problems in the case of very imbalanced classes or with the attraction problem (Sorzano, Semchonok *et al.*, 2021 ▸). In many domains, the procedure has been simplified to separating the data set into training (80–90% of the data) and validation (20–10%) subsets. This is, for example, the case in deep learning, where it is a well accepted practice. This is also the approach suggested by Ortiz *et al.* (2019 ▸).

This article argues that the gold-standard approach is neither necessary nor sufficient to guarantee a lack of bias. It is not sufficient because the two halves can be easily led into the same kind of bias (biased initial volume, missing information induced by the alignment and reconstruction algorithm, incorrect symmetry, the use of incorrect particles, biased objective function, incorrect masking and Fourier filtering *etc*.). If this is the case, both data halves will have the same (or similar) bias. This nonsufficiency argument is well known in the field, in which incorrectly reconstructed structures are reconstructed despite following the gold standard. What is not generally considered in the field is that the gold standard is not necessary either in the sense that processing workflows that systematically interchange images between the two halves do not necessarily show signs of overfitting. This is exemplified in Supplementary Experiment 13. In this example, the set of input images is randomly split at every iteration and assigned to one of the two halves. This strategy is similar to the approach of stochastic gradient descent (Punjani, Brubaker *et al.*, 2017 ▸) with two current solutions instead of one, and it was generalized to multiple solutions in Sorzano, Vargas *et al.* (2018 ▸). In this way, the two reconstructed volumes will surely share common images along the reconstruction history at the end of the processing. Even though this strategy goes against the current belief that total independence of input data is an absolute requirement, we show that there is no obvious sign of overfitting. This experiment is justified by our claim that the overfitting observed in cryoEM is more related to systematic bias than to variance associated with an excessive number of parameters or the lack of independence between the data. In this way, the emphasis in data processing should be more on removing biased parameters rather than the use of half the data for each reconstruction. Still, this practice of constructing two half volumes is useful for calculating the FSC at a particular iteration. This example shows that strategies other than the gold standard are also possible and may appear in the future.

### 5.3. Randomized phases to detect overfitting

Chen, McMullan *et al.* (2013 ▸) suggested the randomization of phases in the experimental images beyond a given frequency as a way to detect overfitting. The calculation of the true SNR in Chen, McMullan *et al.* (2013 ▸) is affected by a problem of zeroth-order Taylor expansion (Sorzano, Vargas, Otón *et al.*, 2017 ▸). This invalidates a faithful calculation of the true SNR based on the FSC of the two halves and the FSC of the two halves after randomizing the phases. In any case, the suggestion makes sense as a characterization of the ability of the 3D reconstruction process to identify overfitting, rather than as a true detector of the overfitting present in the reconstruction without any randomization. A problem with the most used implementation of phase randomization, *RELION*, is that it works at the volume level and not at the level of experimental images as originally suggested. This annuls the whole validation idea. Supplementary Experiment 14 presents the differences between the analysis when the phase randomization is performed at the level of images or volumes. Performing it at the level of volumes does not confirm the validity of the reconstructed volume, and the reported randomized phase FSC is the logical result (a large degree of agreement up to the frequency of randomization and decay from this frequency) of the operation performed.

## 6. Conclusions

The goal of cryoEM is to elucidate the 3D structures of biological macromolecules. This task can be hindered by many pitfalls leading to an incorrect structure determination. The difference between the true (unknown) structure and the obtained structure is our bias. The importance of bias depends on the specific violation and the amount of violation of our data set and parameter estimates. It may affect only small details in the reconstruction or lead to a completely wrong reconstruction. Bias can be induced by the following.

(i) Sample-related sources, such as using particles that do not correspond to our structure, imposing an incorrect symmetry or lacking projection directions. For each of these problems, we have suggested tools that are capable of detecting them and avoiding them if possible.
(ii) Algorithmic related sources such as those related to the initial volume, the particle parameters (angular assignment and in-plane alignment, defocus and acquisition parameters, normalization *etc.*) or the image processing itself (its objective functions or steps inducing bias, in particular masking in real or Fourier space *etc.*). Image-processing biases are hard to fight as they are at the core of the tools available. Still, we should be aware that the image-processing workflow is in itself another source of bias and should do our best to identify incorrectly estimated particle parameters.Generally speaking, we can consider two different kinds of errors when estimating parameters: (1) random errors around the true solution and (2) parameter estimates significantly away from the true solution. Given a single parameter estimation, it is impossible to know which situation we are in. Even if we are given multiple estimates of the alignment and imaging parameters for a single projection, it is impossible to know which situation, (1) or (2), each estimate is in. However, with multiple estimations (at least two), assuming that the algorithms producing them are reasonably correct, we could adopt the following strategy: comparing the estimates and deciding whether most of them agree in some particular region of the parameter space. If this is the case, we may assume that we are in error case (1), and then averaging the parameters would reduce the variability due to each of the estimation processes. If they disagree, we would not know which of the two, or more than two, clusters of parameters is the correct one. We might choose the most populated cluster (if we have more than two estimates of the parameters for the same image), hoping that, since the algorithms estimating them are reasonable, the most populated cluster is close to the (unknown) ground truth. Then, we could average the parameters in that region. We could also ignore those particles for which not all algorithms agree in the parameter region.

Experimentalists are very much aware of sample-related errors, and they try their best to avoid them. Algorithmic errors have been overlooked in the community, and most structures are reconstructed using a single estimate of the particle parameters (a single run of the 3D classification and alignment algorithm), trusting the underlying algorithm always to find the ‘right’ answer and, if not, being capable of dealing with incorrect estimates. Moreover, as a community, we have largely adopted tools (FSC) and strategies (gold standard) that we think protect us from overfitting, that is, structure bias. However, they do not. Unfortunately, there are no statistical means to identify bias without prior knowledge about the reconstructed structure (but this is unknown, as it is the whole purpose of cryoEM). This is a general problem in statistics: bias cannot be estimated from a set of samples.

In this article, we have shown that there might be significant differences between the particle-parameter estimates from different algorithms or even different runs of the same algorithm. Whichever strategy we choose to deal with incorrect estimates at this level, identifying possible bias and reducing the variance invariably requires multiple independent estimations of the alignment and class-membership parameters. Although not entirely protected against bias (two coincident estimates could be simultaneously biased), this approach could help to produce better, more robust and reliable 3D reconstructions of cryoEM data.

## 7. Related literature

The following references are cited in the supporting information for this article: Charbonnier *et al.* (1992 ▸), Chen, Pfeffer *et al.* (2013 ▸), Jaume *et al.* (2001 ▸), Shen *et al.* (2014 ▸) and Thévenaz & Unser (2000 ▸).

## Supplementary Material

Description of Experiments 1 to 14. DOI: 10.1107/S2059798322001978/ic5116sup1.pdf
