# Supplementary material for: On bias, variance, overfitting, gold standard and consensus in single-particle analysis by cryo-electron microscopy
Source: Acta Crystallogr D Struct Biol. 2022 Mar 16;78(Pt 4):410–23. doi: 10.1107/S2059798322001978 (PMC8972802; doi:10.1107/S2059798322001978)
Supplement: Supplementary file 1 [file d-78-00410-sup1.pdf]

STRUCTURAL BIOLOGY

Volume 78 (2022)

Supporting information for article:

On bias, variance, overfitting, gold standard and consensus in single-particle analysis by cryo-electron microscopy

C. O. S. Sorzano, A. Jiménez-Moreno, D. Maluenda, M. Martínez, E. Ramírez-Aportela, J. Krieger, R. Melero, A. Cuervo, J. Conesa, J. Filipovic, P. Conesa, L. del Caño, Y. C. Fonseca, J. Jiménez-de la Morena, P. Losana, R. Sánchez-García, D. Strelak, E. Fernández-Giménez, F. P. de Isidro-Gómez, D. Herrero, J. L. Vilas, R. Marabini and J. M. Carazo

Supplementary Material

Experiment 1. Non-isolated particles.

We selected 32,589 particles from the T20S proteasome of the dataset EMPIAR 10025. These particles were purposefully selected from those having a nearby particle (see Fig. 2). We used as starting volume the correct structure of the proteasome lowpass filtered at 30Å and we imposed D7 symmetry. Relion autorefine reported a resolution of 3.9Å (before post-processing), the same resolution as the one reported for 26,945 isolated particles. In this example, the effect of incorrectly chosen particles has been exaggerated due to the absence of isolated particles ($N_1 = 0$ in Eq. 2) in the dataset. However, it illustrates the general principle that our reconstruction will be a mixture of a correct and incorrect structure. Depending on the proportion of incorrect particles, the incorrect structure will be more or less visible on top of the correct structure. In any case, it will be biasing our result.

Experiment 2. 3D classification stability.

In the following, we describe the results of three different classification experiments:

1. Absence of heterogeneity. We selected 24,199 particles from the Brome mosaic virus sample of EMPIAR 10010. We used Relion to separate these images into two classes (the two classes were internally initialized by Relion by randomly assigning the input images to one of the two classes, which is the most common way of executing Relion 3D classification). We used the 3D reconstruction of all these particles as initial volume, which yielded a reconstruction of 4.1Å resolution. During the classification, we imposed the icosahedral symmetry. We repeated this step three times, and the number of particles yielding the virus structure ranged from 68.4% to 82.8%. In all cases, the remaining particles gathered into a class in which there was a mixture of several kinds of misalignment (including mirroring of the particles). Only 55.8% of the images were always assigned to the correct virus structure in all three classifications, meaning that the class assignment was unclear for 45% of the particles. This result is very interesting because it shows the difficulty of determining the class parameter, even for an easy example in which there is a single class.
2. Continuous heterogeneity. We selected 34,268 particles from the ribosome sample of EMPIAR 10028. The head of this ribosome is moving and we submitted the set of particles to a classification with Relion in three classes. We used as initial volume the reconstruction of all the particles at 4.4Å and imposed no symmetry. We repeated this classification three times obtaining the following class distributions:

Run	Class 1	Class 2	Class 3
Run 1	46.3%	46.0%	7.7%
Run 2	37.2%	31.8%	31.0%
Run 3	40.1%	31.1%	28.8%

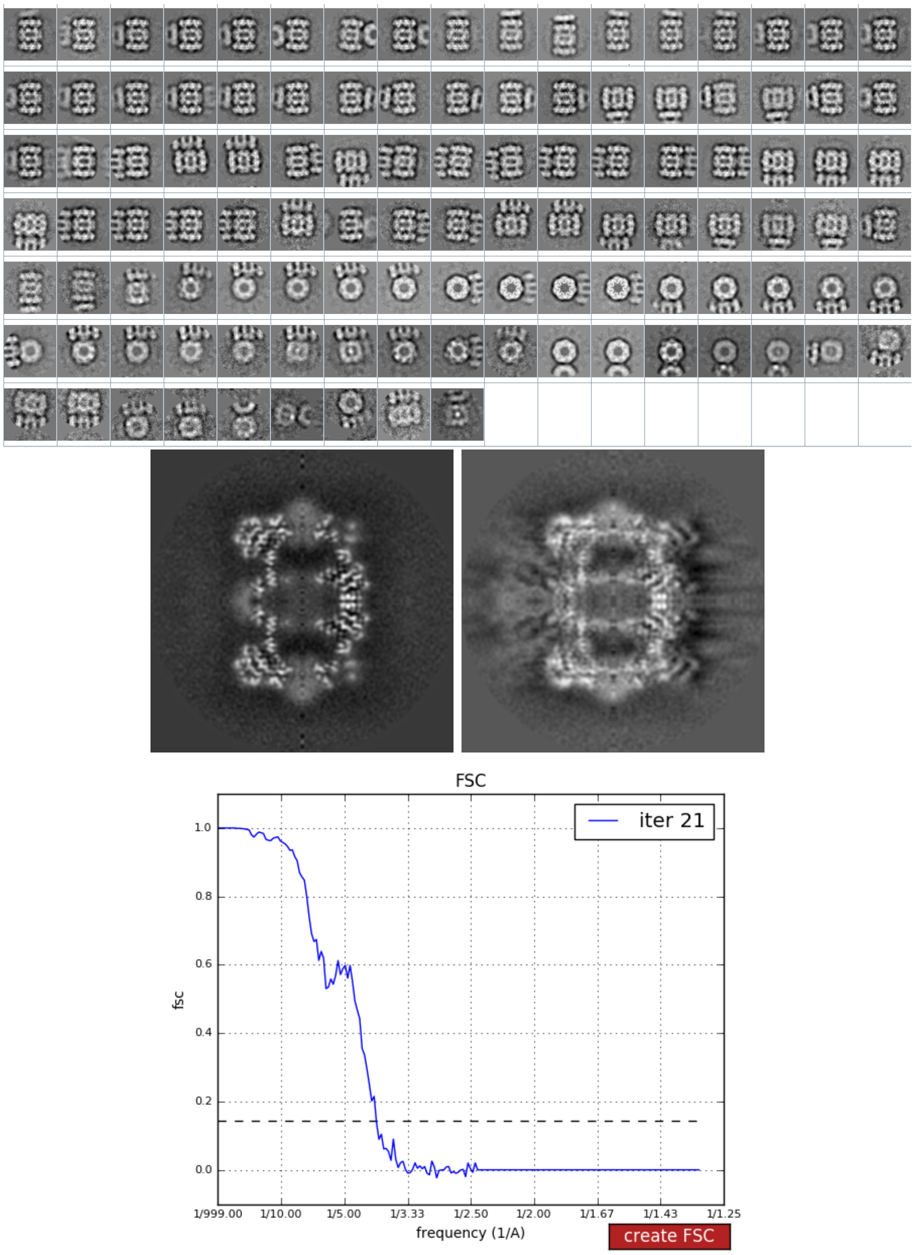

Figure 2: (This Figure corresponds to Experiment 1.) Top: Gallery of the 2D class averages of the particles selected for 3D reconstruction. Middle: Central slice of the 3D reconstruction with isolated (left) and joint (right) particles. Bottom: FSC of the reconstruction with joint particles.

If we compute the subsets of particles that were always classified together, one might expect that in a stable classification, most of the particles in a class stay together independently of the classification run. There are up to 27 ($= 3^3$) possible subsets, of which 19 have sizes of 8.0%, 6.8%, 6.0%, 5.8%, 5.8%, 5.6%, 5.6%, 5.2%, 5.2%, 5.1%, 4.9%, 4.7%, 4.3%, 4.3%, 4.0%, 3.9%, 3.9%, 3.7%, 3.6%, and 8 small classes have less than 1% each. This means that the three classifications are not really consistent with each other. The input particles are almost "uniformly" scattered among the three classes even though true classes could have been formed due to the head movement.

3. Discrete heterogeneity.
   - Case 1. 10,000 images of the Ribosome 70S subunit has served for years as a benchmark for 3D classification [69]. The first 5,000 images of this dataset are supposed to belong to one class and the last 5,000 to another class. We used as initial volume the reconstruction using all particles (with a resolution of 13.5Å). We performed a 3D classification of this dataset into two classes using Relion. Most of the images were assigned to a large class (containing 87-89% of the particles), while the remaining images were assigned to a small class. 85% of the original particles were always classified together. This may indicate two possibilities: a) these 8,500 images really belong to a single class (instead of the 5,000 originally assumed); b) the larger class attracts particles from the smaller class by an effect of 3D attraction. With experimental data, it is not possible to distinguish the true underlying state of the class labels. Still, the dataset has been used in the field several times as a 3D classification benchmark.
   - Case 2. The EMPIAR entry 10333 contains 23,307 images of Human FACT in Complex with Partially Assembled Sub-nucleosomes. The original authors classified these images into two classes of 16,317 (70% of the dataset) and 6,990 (30%) images, respectively. We repeated the classification in two classes five times using Relion. We used the reconstruction performed by the original group as the initial volume (EMDB entry 20840, 4.9Å). In each one of the runs, the largest class obtained: 58%, 71%, 87%, 92%, and 93% of the dataset. This means that the two classes were not separated in the last three of the five runs (another manifestation of the 3D attraction). Once a class starts increasing its SNR, it attracts all particles, disregarding whether they truly belong to that class or not). In the first two runs, the presence of the original classification in the recalculated classification is only between 60-65% (a little bit lower than the 70% that one would expect from a random classification; and much lower than the 100% for a classification that matches the original one). If we analyze the set of images that were consistently put together into the same class, the decomposition of the original dataset is: 43%, 19%, 15%, 7%, and many small groups with less than 2% of the images. This experiment

shows how difficult 3D classification is in such noisy environments, and it suggests that just taking “the first classification result” is not a safe strategy.

Experiment 3. 3D Angular attraction caused by overrepresented directions.

We simulated 36,000 projections of the EMDB entry 10077 (Erythromycin Resistant *Staphylococcus aureus* 50S ribosome (delta R88 A89 uL22) in complex with erythromycin). The structure was solved to 2.3Å. 10,000 of these images were randomly distributed over the projection sphere, while the rest were concentrated in a cone of 30 degrees. We added noise to an SNR of 0.1. We ran Relion autorefine starting from the already solved structure. We used C1 symmetry, and Relion automatically determined the number of iterations. The isosurface of the resulting reconstruction by Relion autorefine is shown in Fig. 3. It can be seen that there is a strong elongation along the overloaded (vertical in the figure) direction caused by the attraction of that direction of nearby particles (the non-overloaded particles had a probability of being found in this region that was 50% larger than expected by a uniform angular distribution). An angular distribution analysis revealed that the 3D attraction did not cause any missing region in this case. However, it attracted to the over-represented region many images that did not belong there (see the histogram in Fig. 3).

Experiment 4. Different angular assignments.

We selected 15,396 images of the β-galactosidase sample of EMPIAR 10013. We used an initial volume calculated by Xmipp `reconstruct_significant` [83] exploiting the D2 symmetry of the macromolecule. We reconstructed it with Xmipp highres and Relion autorefine. The resolution calculated by the FSC at 0.143 was 2.9 Å and 3.9Å, respectively (the reported resolution at EMDB was 3.2 Å; we report the resolution calculated without postprocessing). When we compare the angular assignment of both algorithms, we observe that there is a wider empty region in the case of Relion and that 48% of the particles obtain an angular assignment significantly different between Xmipp highres and Relion autorefine (see Fig. 4), meaning that at least one of the two angular estimates has to be wrong for the particles in which both algorithms do not coincide.

Experiment 5. Angular assignment stability.

We selected 34,268 particles from the ribosomal dataset at EMPIAR 10028. We used as initial volume the reconstruction of all the particles at 4.4Å and imposed no symmetry. We performed five independent angular assignments using Relion autorefine (Relion automatically determined the number of iterations). Between any two angular assignments, about 7% of the particles obtained a significantly different angle (larger than 1.5 degrees in a structure whose size is 400 voxels wide, that is an uncertainty larger than 5 pixels in the border of the image). Only 88% of the particles consistently obtained an angular assignment whose angular difference was smaller than 1.5 degrees.

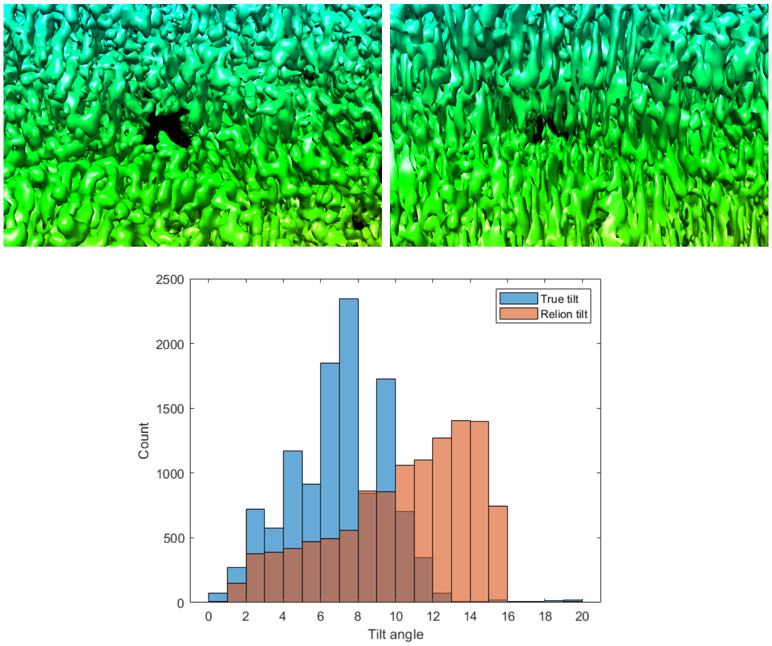

Figure 3: (This Figure corresponds to Experiment 3.) Top: Isosurface of the 50S ribosome in complex with erythromycin reconstruction when a particular direction is overloaded reconstructed by Xmipp highres (left) and Relion 3D autorefine (right). The surface has been colored as a way to help visualization (according to the volume height). Bottom: Ground-truth tilt angle distribution and its estimated distribution by Relion 3D Autorefine.

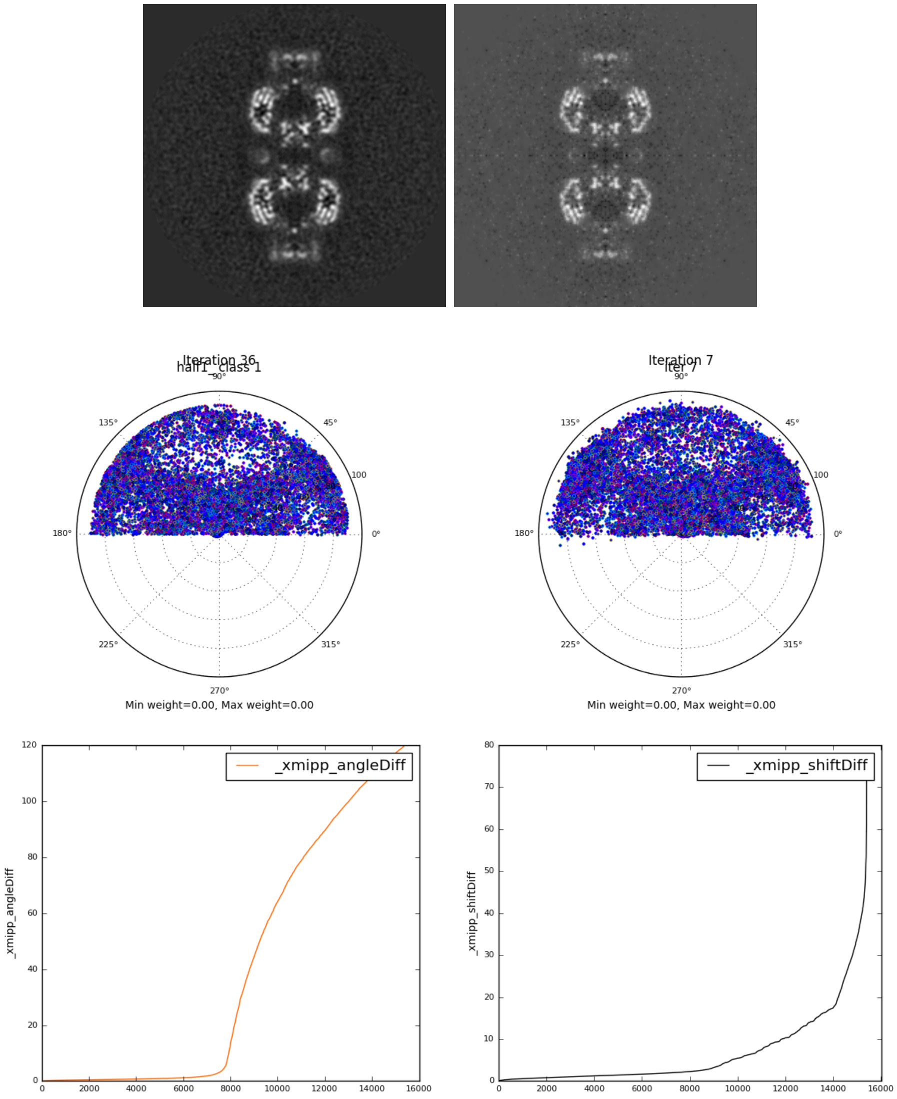

Figure 4: (This Figure corresponds to Experiment 4.) Top row: Central reconstruction slice by Relion autorefine (left) and Xmipp highres (right). Middle row: Angular distribution of Relion (left) and Xmipp highres (right). Bottom: Comparison of the angular difference (left) and shift difference (right) between both algorithms (note that these figures are not histograms but a plot of the sorted values).

The same experiment performed with 26,945 projections of isolated the T20S dataset of EMPIAR 10025 revealed that only 62% of the particles obtained an angular alignment within 1.5 degrees when two independent angular assignments by Relion autorefine were compared. For that experiment, we used an initial volume obtained by Xmipp `reconstruct_significant` [83], lowpass filtered at 30Å. We imposed D7 symmetry.

These experiments show that the uncertainty of the angular assignment is not only due to the use of different assignment algorithms (see Experiment 4) but also due to an uncertainty intrinsic to each algorithm and dataset.

Experiment 6. Different similarity measures.

We analyzed the 26,945 T20S particles of the previous experiment. We plotted the histogram of the Log-Likelihood and the maximum probability as calculated by Relion autorefine. Neither of the two plots reveals the presence of two subpopulations (a population of correctly assigned particles and a population of incorrectly assigned particles or non-particles, for instance), see Fig. 5. However, when we compute the Xmipp highres local similarity (the cross-correlation within a maximal circle), the existence of the two populations can be easily recognized (see Fig. 5). This example illustrates that a single metric can measure only one feature of the data being analyzed. Different metrics allow us to see the dataset from different perspectives. In some of these perspectives, data pathologies like the presence of multiple populations of particles may be more visible than in others (i.e., in other data sets, the difference of behavior in the metric that in this case favors Xmipp highres could have played the other way around and favor Relion). Additionally, we split the input dataset into two groups depending on the low or high local cross-correlation. Performing two reconstructions with these two groups shows that the FSC of one of them is much better than the other one, indicating that one of the groups, the one with low local correlation, had been incorrectly aligned by Relion 3D autorefine (see Fig. 5).

In Maximum-likelihood or Maximum a Posteriori methods, particles can have different angular assignments, each one with a different probability. In the best case, it is expected that the probability distribution for a particle converges to a delta function so that the particle has a unique angular assignment. However, this is not the general case, and a particle usually has several orientations with a probability significantly different from zero. This is a direct consequence of the metric being used and its smoothness. To illustrate this effect, Fig. 6 shows the distribution of the number of significantly contributing alignment parameters in Relion autorefine for these experimental images.

Experiment 7. Handedness mixture.

We selected 26,945 particles of the proteasome dataset EMPIAR 10025. We generated an initial volume that had mirrored and unmirrored 2D classes mixed (we did so by mirroring the initial volume of Experiment 5 and adding it to itself). We then refined this initial model with the particles using Xmipp highres.

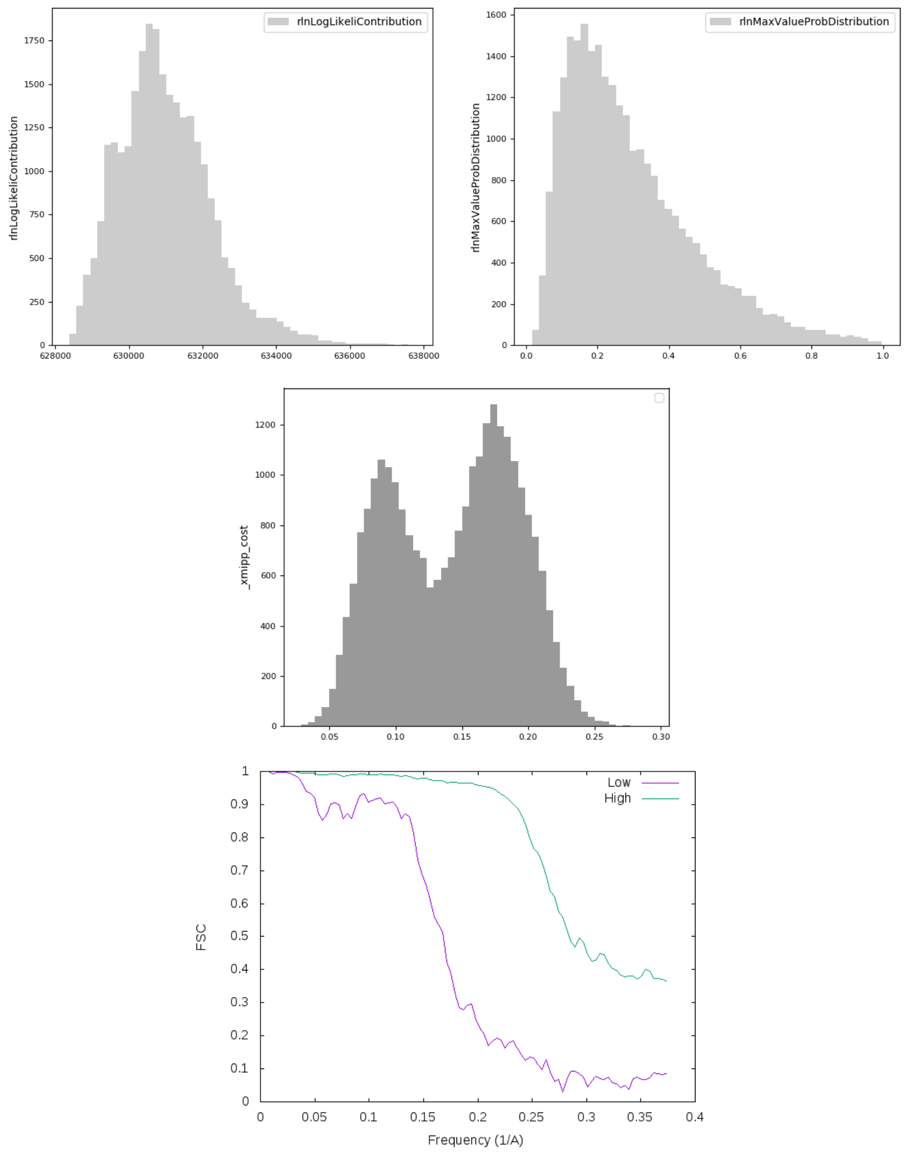

Figure 5: (This Figure corresponds to Experiment 6.) Top: Histogram of the log-likelihood (left) and maximum probability (right) of Relion autorefine. Middle: Histogram of the cross-correlation index within a maximal circle. Bottom: FSCs of two reconstructions performed with the particles in the low and high local correlation groups.

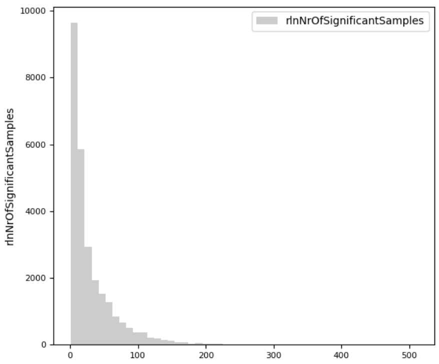

Figure 6: Histogram of the number of significantly contributing alignment parameters for Relion autorefine.

We performed a global angular assignment, but projection matching starting from an initial model (even with all the safeguards built in highres) is, by nature, a local optimization in the volume space. As shown in Fig. 7, the refined volume keeps the hand mixture resulting in an incorrect map. Since the hand mixture is present in both halves, the FSC reports a resolution of 4Å at a threshold of 0.143. If the multiresolution reconstruction scheme of highres is employed, then the algorithm converges to the right structure. Solving the problem at low resolution helps to smooth the landscape of solutions in the volume space and helps the algorithm find a better minimum. This smoothing of the goal function is an effect that has already been reported in other image processing domains like image registration [94], image segmentation [28], or image restoration [10]. However, note that there is no guarantee that by smoothing the goal function, the algorithm will be able to find the global minimum starting from any point.

Experiment 8. Incorrect CTF correction

We selected 22,824 particles from the proteosome dataset EMPIAR 10013. We constructed the initial volume in the same way as in Experiment 4. We reconstructed it with Relion autorefine obtaining the reconstruction shown in Fig. 8a. We then reduced all the defoci of the particles by -0.5μm (this corresponds approximately to 20% of the average nominal defocus). The corresponding reconstruction (Fig. 8b) has a dark halo surrounding the particle (this halo is often observed in public EM maps). The problem is not only the halo itself but the fact that there are also structural differences between reconstructing using the correct defoci and the incorrect defoci, as shown in Fig. 8c. The difference between the reconstruction with the supposedly correct defoci and the ones with the systematically wrong defoci is a structural bias that is superposed to our final 3D reconstruction. However, the FSC reported the same resolution, 3.8Å, for the incorrect and correct defocus experiment.

Errors in the CTF correction can also be induced by incorrect pixel size since the CTF formula depends on it, too, as shown in the main text. In

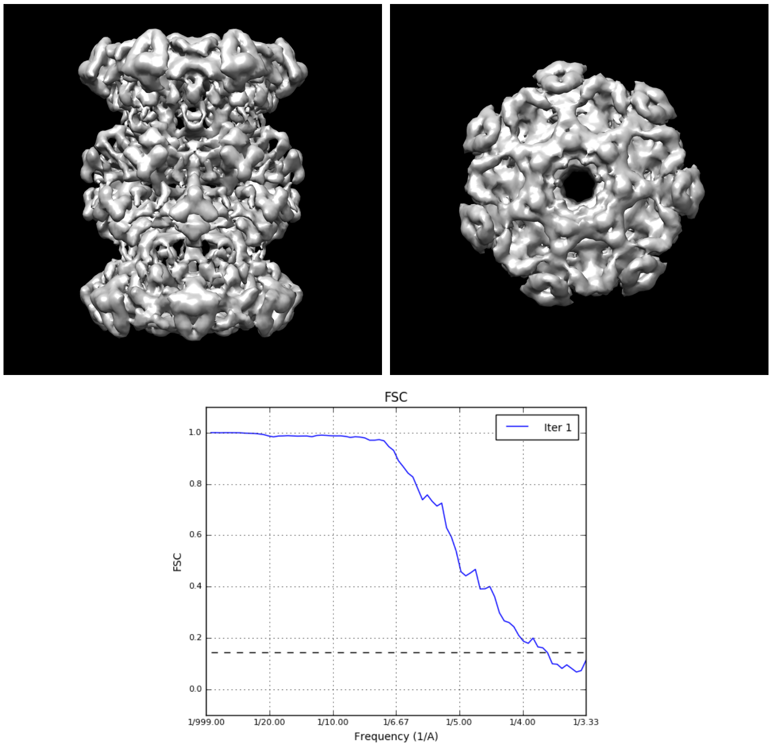

Figure 7: (This Figure corresponds to Experiment 7.) Top: Side and top views of a proteasome dataset when the initial volume has a hand mixture. Bottom: FSC of the reconstruction according to Xmipp highres. Despite the seemingly correct FSC, the reconstruction is totally artifactual as half of the images have been mirrored. Consequently, we have the addition of two volumes: one with the correct hand and another one with the incorrect hand.

our next experiment, we systematically reduced the pixel size by a factor of 20%. Although these large errors are not expected in a real experiment, it illustrates the problem and, in practice, several algorithms like Xmipp highres [82] or cisTEM [20] can optimize for the magnification at the level of particle and, if not properly constrained or monitored, pixel size deviations as large as 20% can be observed for some images. The FSC reported in this case a resolution of 3.1Å (actually, the FSC frequency axis is itself heavily affected by a biased pixel size; the reason is that the Fourier index (k_x, k_y, k_z) of a volume of size $N_x \times N_y \times N_z$ voxels is translated into a continuous frequency given by $(f_x, f_y, f_z) = \left(\frac{k_x}{N_x T}, \frac{k_y}{N_y T} \frac{k_z}{N_z T}\right)$, where T is the pixel size in Å/pixel, in this way, errors in the pixel size are translated into errors of the frequency axis of the FSC and, because of the inverse relationship, errors in which the pixel size is underestimated are amplified by the inverse relationship). Fig. 8 shows how the FSC is, in this case, misleading in order to distinguish a correct from an incorrect reconstruction.

Experiment 9. Volume postprocessing

We selected 34,268 from the ribosomal dataset EMPIAR 10028 (in a similar way to Experiment 5). We reconstructed it with Relion autorefine and post-processed it with Relion. The FSC is shown in Fig. 9. As argued in the text, the change in FSC is solely due to the change in the mask between the 3D reconstruction and the post-processed one. Masking is another source of bias, this time with a more visible effect in Fourier space. In Sec. 5.1 we discuss that a bias in the volume results in arbitrarily high FSCs.

Relion's implementation of the postprocessing also reports the Phase Randomized FSC and its correction. However, the randomized maps FSC is performed at the level of volumes (the phases of the volumes are randomized beyond a given frequency), giving a false impression of reliability on the result obtained from the 3D reconstruction process (the corrected FSC is also affected by the bias induced by the masking). The randomization should be performed at the level of images, as originally suggested by [11], and then the whole 3D reconstruction and alignment procedure should be repeated (see Suppl. Material Experiment 14). In this example, the resolution estimated by the corrected FSC was 3.53Å, while the resolution estimated by the unmasked maps (as reported by Relion autorefine) was 4.54Å. To ascertain which of the two numbers was closer to the underlying resolution, we independently measured local resolution with MonoRes [101]. The histogram of local resolutions is also represented in Fig. 9. We can see that the peak of local resolution is around 4Å and that between 4-5Å there is a large fraction of the voxels.

Experiment 10. Algorithm weight

We selected 10,908 particles from the ribosomal dataset EMPIAR 10028 (the initial steps of the processing are the same as in Experiments 5 and 9). We reconstructed it with Relion autorefine obtaining the reconstruction shown in Fig. 10 left. Using the same angular assignment within Relion reconstruct,

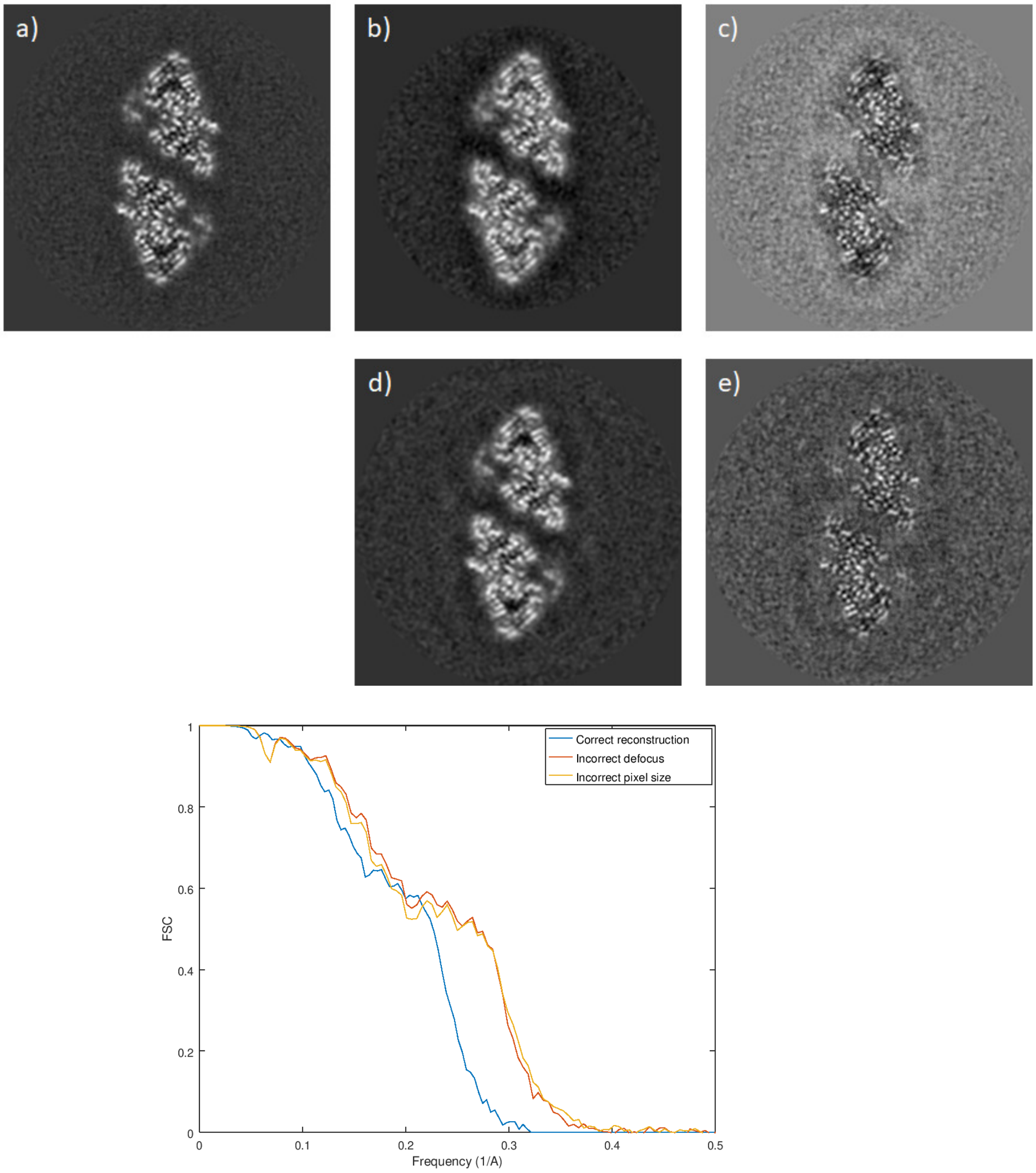

Figure 8: (This Figure corresponds to Experiment 8.) Representative slices of a reconstruction with the correct CTF (a), a reconstruction with biased defocus (b), the difference between a and b (c), a reconstruction with biased pixel size (d), and the difference between a and d (e). Bottom: FSC of the correct reconstruction and the reconstruction with incorrect defoci and incorrect pixel size.

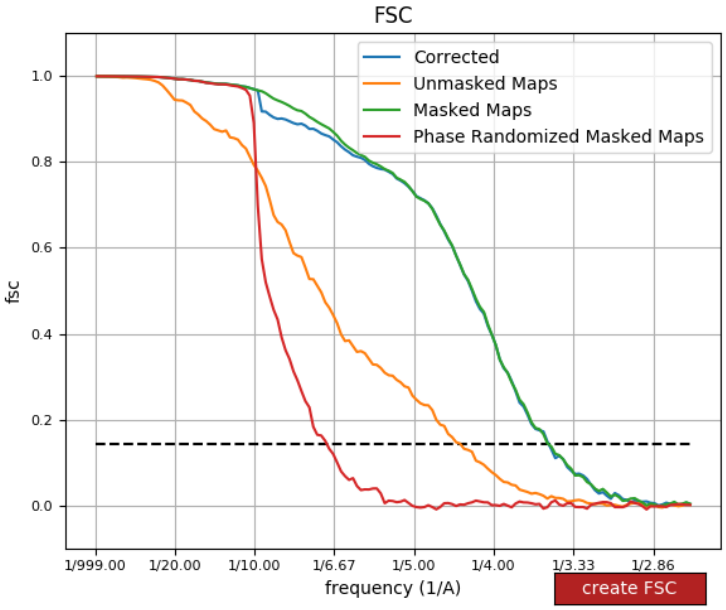

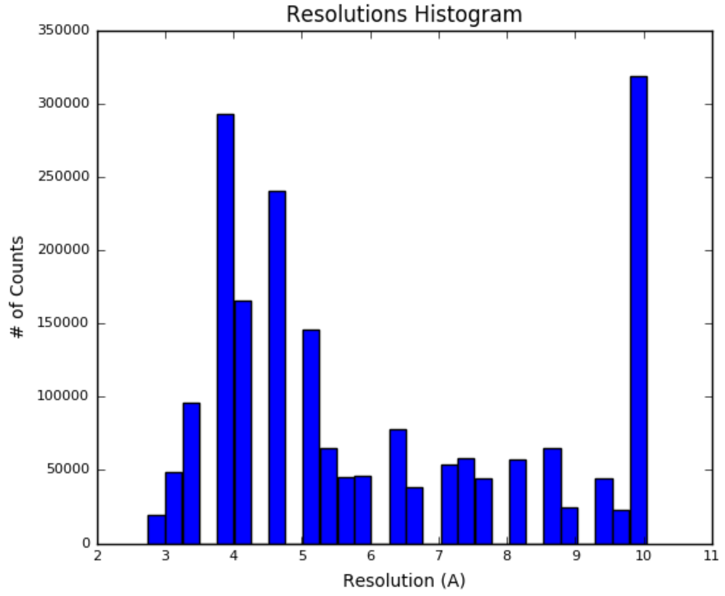

Figure 9: (This Figure corresponds to Experiment 9.) Top: Different FSCs reported by Relion: Unmasked (just coming out from the reconstruction process), Masked (after changing the mask), Corrected (after changing the mask and B-factor corrected), Phase Randomized at the volume level (after randomizing the two half maps from 10Å). Bottom: Histogram of the local resolution as calculated by MonoRes.

we obtain the reconstruction shown in Fig. 10 right. The difference between the two volumes is a bias between both reconstructions. The only difference between these two reconstructions is the internal weight assigned to each of the projections, each of the frequencies, and the fact that in autorefine, the same particle can occupy multiple locations (these weights are the ones at Eq. (9) of Scheres [62]). This example illustrates the fact that the 3D reconstruction algorithm itself is another source of bias.

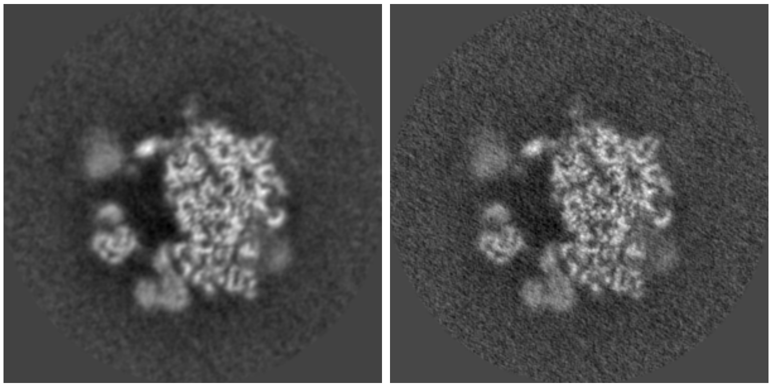

Figure 10: (This Figure corresponds to Experiment 10 .) Central slice of the ribosome reconstructed with Relion autorefine (left) and Relion reconstruct (right).

Experiment 11. Effect of non-linear processing on the FSC

We converted the atomic model of the Peripheral domain of open complex I during turnover (PDB entry: 6ZK9) into an electron density map using electron atomic scattering factors [85]. We then added two realizations of Gaussian noise with zero mean and standard deviation 0.3 (see Fig. 11). These two volumes would be the equivalent of the two half maps from the gold standard approach. Then we calculated the average of the two half maps and the non-linear processing of Xmipp highres (it includes denoising in real space, filterbanks, denoising after applying a Laplacian transformation, deconvolving, applying a soft-negative undershooting remover, and a weight based on the pair differences, see the Suppl. Material of [82] for the algorithmic details). Note that the non-linear processing does not involve any masking (although a mask is used to define a region in which noise statistics can be measured) and that the concept of statistical significance is very much involved in all steps.

We then compared the FSCs coming from linear and non-linear processing. As expected, the 0.143 threshold is a good estimate of the resolution of the two gold-standard maps (see Fig. 12 top). It effectively represents the resolution when the average of the two maps is compared to the atomic model. Interestingly, if we remove the surrounding noise with a relatively generous mask (see Fig. 11), the FSC is largely boosted. However, the map has not been touched, and it could be rightly argued that the map's resolution is the one after masking

(as we have only removed noise that is known not to belong to the structure). At this point, we do not enter into the issue of the right resolution value and only highlight the dependence of the FSC on two reasonable approaches to its calculation.

We next applied the series of non-linear processing steps described above and used by Xmipp highres. We compare the gold standard FSC to the FSC of the highres map to the atomic model (see Fig. 12 middle). For comparison purposes, we also included the comparison of the average of the two half maps (linear processing) to the atomic model. The FSC of the highres gold standard shows that the non-linear processing of the two half maps is extremely consistent (the FSC does not even cross the 0.5 threshold). By comparing the highres result (defined as the average of the two non-linearly processed half maps) to the atomic model, we can estimate whether these restored maps have introduced consistent but incorrect features. From this comparison and the corresponding one of the linear processing, we see that the non-linear processing outperforms the linear one. We wondered then if the improvement was due to an implicit masking effect of the non-linear processing, and for checking that, we applied the same mask to the linearly and non-linearly processed maps. We then compared the masked volumes to the atomic model (see Fig. 12 bottom). From the FSC, we can see that the improvement is not only due to masking, but there are improvements inside the mask too.

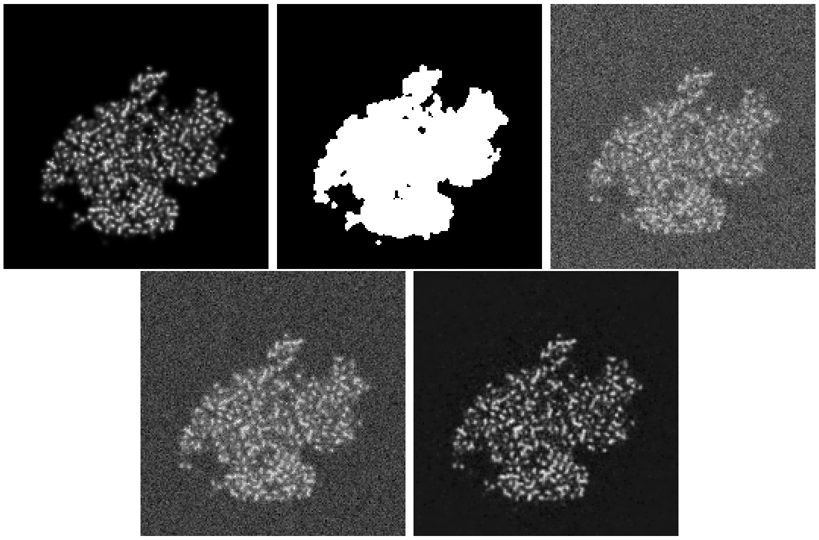

Figure 11: (This Figure corresponds to Experiment 11.) From top to bottom, left to right, slice 84 of: the atomic model, the mask, one of the half maps, the average of the two half maps, and the non-linearly processed map.

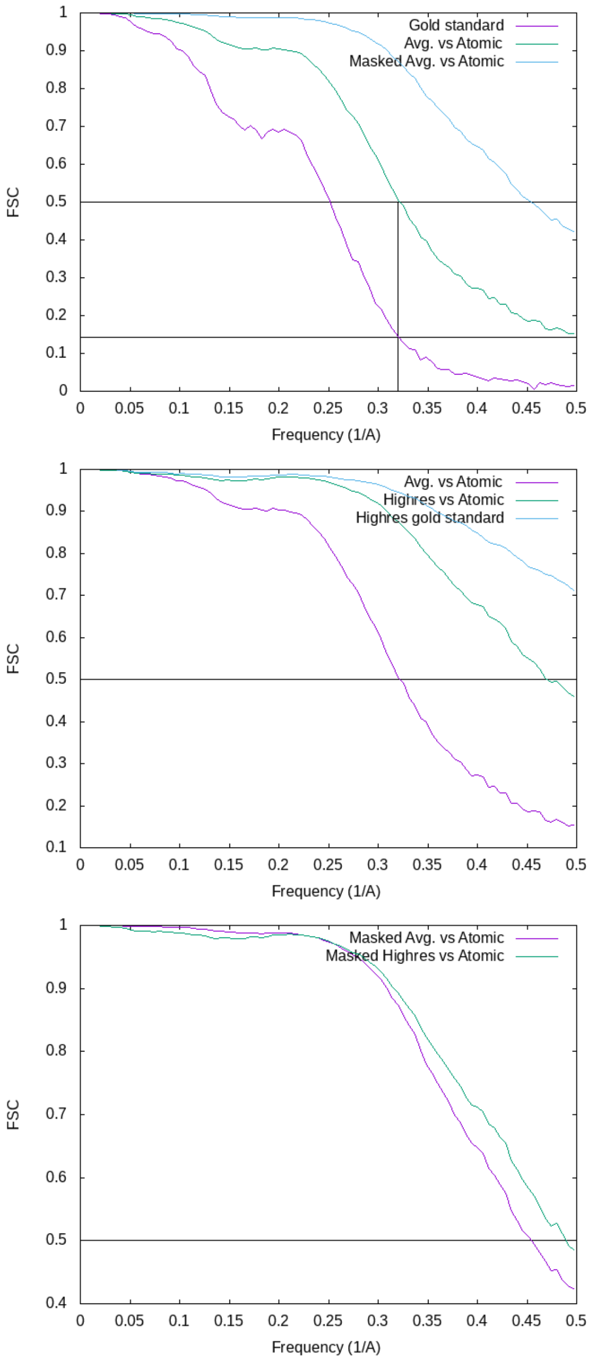

Figure 12: (This Figure corresponds to Experiment 11.) Top: FSC corresponding to linear processing. Middle: FSC corresponding to non-linear processing. Bottom: Comparison of the FSCs between linear and non-linear processing after masking.

Experiment 12. Change of regime of the FSC

We selected 34,268 particles of the ribosome dataset EMPIAR 10028 (the initial volume was constructed as in Experiment 5). We filtered them with a lowpass filter starting to decay at 5Å and totally vanishing at 4.6Å with a raised cosine decay in between. We applied 6 iterations of Xmipp highres with an increasing target resolution (this algorithm is multiresolution, and it downsamples the input images to a pixel size such that the target resolution is at 2/3 of the Fourier frequency range; this is the reason why the FSCs of the different iterations, shown in Fig. 13, finish at different frequencies). In this plot, we see that at 4.6Å there is a clear regime change in the FSC for highres. The same change is observed in Relion's autorefine (this time at 4.8Å). The comparison of the two maps (highres and Relion; see slices in Fig. 13) reveals that there are two fundamental differences: 1) the noise outside the molecule is practically inexistent in highres (there is no hard masking, but there is an automatic detection of the statistical distribution of the noise under several transformations) [82], and a dampening by significance in the whole volume); 2) the details of highres inside the molecule are finer than in Relion. Remember also that the FSC at a given frequency can be understood as the correlation in real-space of two bandpass filtered volumes at that frequency [86]. The fact that the FSC on the left does not go to 0 simply indicates that the two volumes have a medium correlation to each other, disregarding their absolute amount of energy. Actually, if part of the postprocessing dampens the noise at high-frequency, then the two half-maps at high frequency may correlate better despite having much less energy due to the dampening.

This experiment illustrates the fact that the 0.143 FSC threshold is based on an assumption of linearity of the 3D reconstruction process that the 3D reconstruction algorithm may not fulfill (in particular, 3D Fourier gridding is not linear, nor are the noise removal steps of highres; additionally, although there is no explicit masking, the noise reduction of highres results in a smoothing of the solvent outside the particle, see the slice of Fig. 13, that is a soft mask that induces correlation at high frequency). Rather than a specific threshold, the resolution of a map is better determined by a change in the FSC behavior. However, it is more difficult to give a formal criterion to detect this point.

Experiment 13. The gold standard is not necessary

We selected 16,703 particles of the brome mosaic virus dataset EMPIAR 10010. The initial volume calculation was performed as in Experiment 3. We ran Xmipp highres following the gold standard strategy of splitting the dataset into two independent halves that never share information over the iterations. The results of this execution are shown in Fig. 14. We also ran Xmipp highres with a stochastic split at each iteration (9 iterations were performed). At each split, each experimental image may go to either half so that the halves are not independent anymore over iterations. As shown in Fig. 14, there is no significant difference between the two strategies. Icosahedral symmetry was used during the reconstruction. Similar results have been obtained with other macromolecules with lower symmetry (even C1).

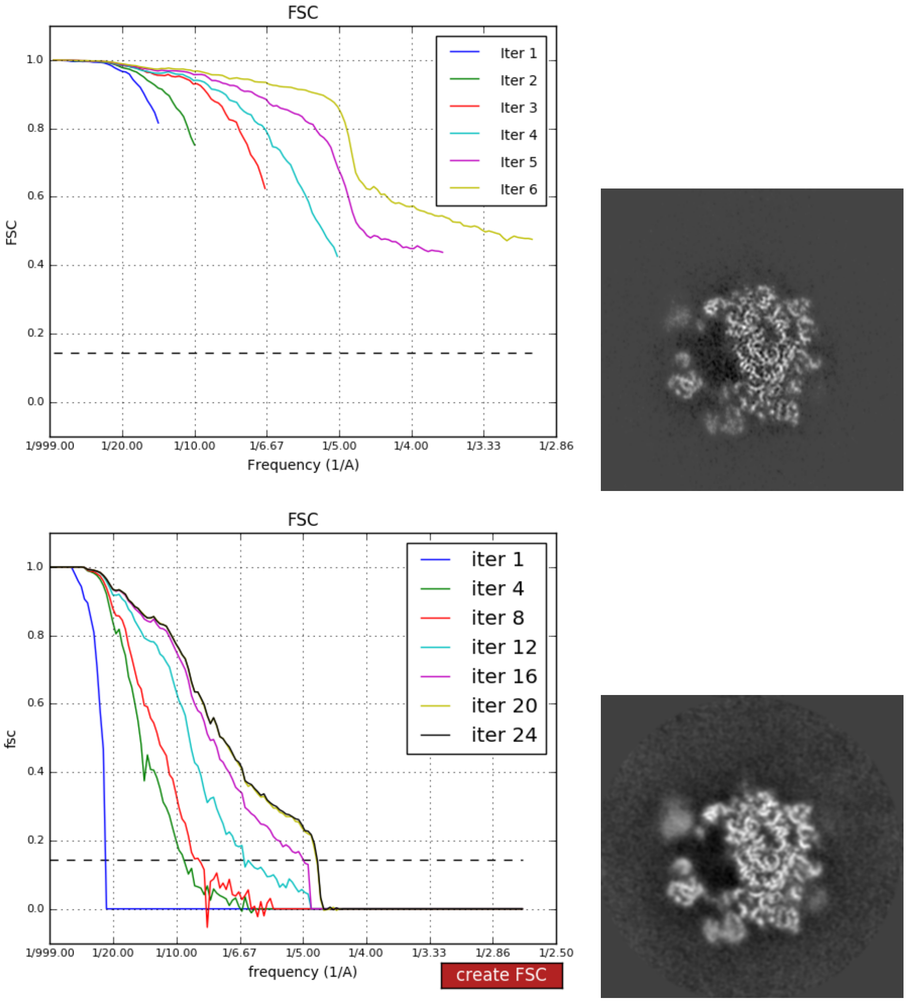

Figure 13: (This Figure corresponds to Experiment 12.) Top row: FSCs and a central slice of Xmipp highres when the input data is filtered at 5Å, the filter fully vanishes at 4.6Å. Bottom row: Same information for Relion autorefine.

This experiment shows that there is not a strict need to separate the dataset into two independent halves. Overfitting can also be avoided if specific measures are taken during the 3D reconstruction process (in the case of highres, the projection weighting scheme depending on the confidence on its angular assignment and the noise attenuation steps performed after reconstructing).

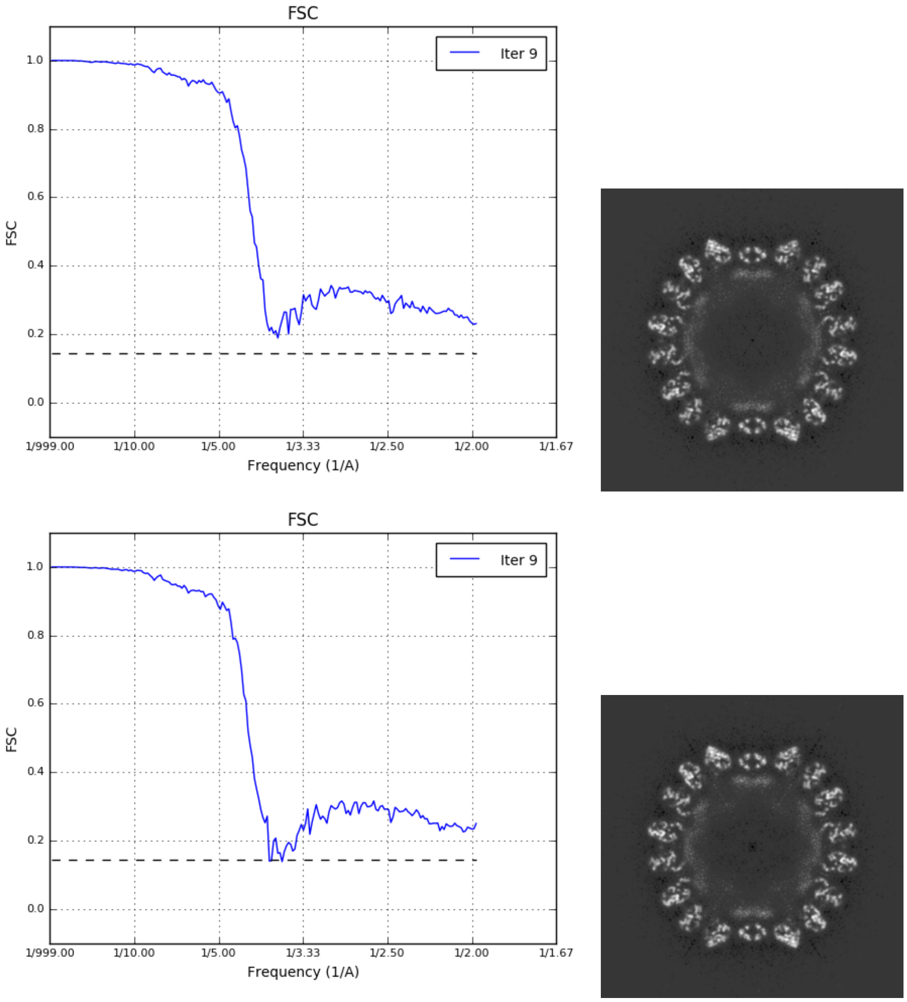

Figure 14: (This Figure corresponds to Experiment 13.) Top row: FSC and a central slice of Xmipp highres running the gold standard. Bottom row: FSC and a central slice of Xmipp highres running in stochastic split mode.

Experiment 14. Phase randomization

We selected 34,268 particles of the ribosome dataset EMPIAR 10028 (the initial steps of the processing were like in Experiment 3). We randomized the

phases of these particles beyond 5Å and performed a 3D angular assignment and reconstruction using Xmipp highres and Relion autorefine. In both cases, we observe the change of FSC regime exactly at 5Å as expected, meaning that both algorithms do not overfit the Fourier components beyond 5Å. You may compare the shape of these FSC curves obtained when images are phase randomized and those obtained when the randomization is performed at the level of volumes (see Suppl. Material Experiment 9). The alignment of the phase randomized images using Relion autorefine took more than 7h using 2 GPUs. In contrast, the phase randomization curve calculated by Relion postprocessing took 17s (this time difference shows why practitioners prefer the result from the postprocessing).

This experiment shows that the FSC obtained with phase randomization at the level of images (Fig. 15) is not the same as the FSC obtained at the level of volumes (Fig. 9). Note that if the phase randomization is performed on the images, the high frequencies of the images cannot contribute to determining the alignment of the particles. However, if the phase randomization is performed only on the volumes, the high-frequency components of the images have contributed to determining better estimates of the angular orientation of the images, and the phase randomization loses its validation power.

Additionally, at the level of volumes, there is an overestimation of the FSC randomized (see that in Fig. 9, the phase randomized FSC crosses the standard 0.143 threshold at 6.6Å, instead of 10Å that is where the phase randomization in the images really started, note that this number 10 was arbitrarily chosen for the purpose of this particular example). This overestimation of the phase randomized FSC is later translated into an overestimation of the corrected FSC through Eq. (4) of Chen et al. [12].

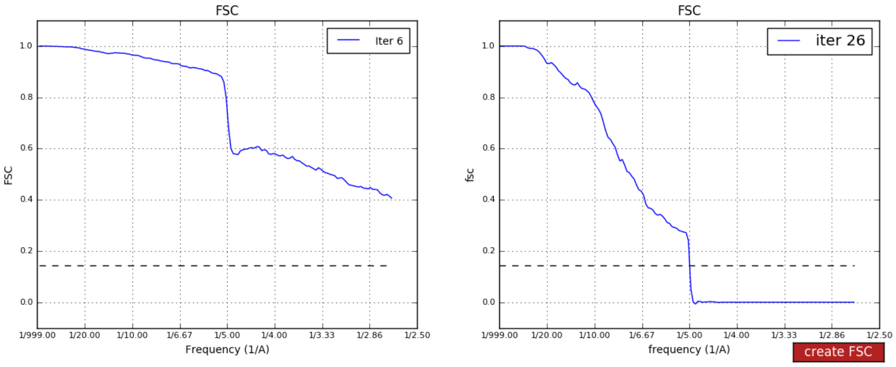

Figure 15: (This figure corresponds to Experiment 14.) FSC of Xmipp highres (left) and Relion (right) after randomizing the phases of the experimental images beyond 5 Å.
